# Photo-Patternable Organic Electrochemical Transistors with Hydrophilic and Hydrophobic Bulk Heterojunction Enabled by Ethylene Glycol-Based Photo-Crosslinker

**DOI:** 10.3390/polym18172057

**Published:** 2026-08-25

**Authors:** Gu-Hao Cai, Yun-Cheng Guo, Sin-Rong Huang, Po-Hsiang Fang, Jung-Yao Chen

**Affiliations:** 1 Department of Photonics, National Cheng Kung University, Tainan 70101, Taiwan; 2 Academy of Innovative Semiconductor and Sustainable Manufacturing, National Cheng Kung University, Tainan 70101, Taiwan; 3 Meta-nano Photonics Center, National Cheng Kung University, Tainan 70101, Taiwan

**Keywords:** organic electrochemical transistor, photolithography, response time

## Abstract

Organic electrochemical transistors (OECTs) utilize ion injections to regulate the overall conductivity of the organic semiconductor channel, achieving high transconductance (*g*_m_) by coupling ionic and electronic charge carriers within the whole channel’s volume. However, the slow ion migration rate through the hydrophobic semiconducting polymer layer restricts the response rate of the device for widespread applications in biomedical sensing. This work introduced poly(ethylene glycol) (PEG) bisazide photo-crosslinking agent into the p-type semiconducting polymers as a hydrophilic active channel in accumulated-mode OECTs. Upon incorporation of PEG segments into conjugated polymers via photolithography, the resulting OECTs exhibit a significant enhancement in both µC* product and doping/de-doping dynamics by at least one order of magnitude. The photo-patterning of an ion-conducting semiconductor channel with a minimum line gap of 5 µm enables the fabrication of a depletion-mode inverter. This study presents a straightforward patterning process that enhances the hydrophilicity of various hydrophobic conjugated polymers while eliminating the need for complex synthesis procedures typically required for introducing ethylene glycol side chains on conjugated polymers.

## 1. Introduction

π-conjugated polymers have diverse applications, including optical displays, energy storage, neuromorphic computing, and bioelectronics. They offer advantages like solution processability, mechanical flexibility, and compatibility with living systems [1,2,3,4,5,6,7,8]. In recent times, there has been extensive attention towards the advancement of organic electrochemical transistors (OECTs) in the realm of bio-electrochemical devices such as biomedical sensing, digital logic, and neuromorphic devices [9,10,11,12,13]. OECTs rely on simultaneous ion and electron transport during electrochemical doping [14,15,16,17]. Proper electrolyte formulation is crucial for device performance, especially in biological settings that require rapid and reversible ion transport. Considering the hydrophobic conjugated polymer backbone for electronic charge carrier transport, achieving the right balance in hydrophobic–hydrophilic profile is essential for optimal bio-electrochemical device performance [14,18].

Due to the ionic charge transport nature of OECTs, the improvement in hydrophilicity in conjugated polymers plays a crucial role in enhancing the overall device performance. The most employed method involves introducing polar ethylene glycolated side chains, which effectively enhances the conjugated polymer’s compatibility with aqueous environments. Since its initial demonstration in the early 1990s [18], similar strategies have been extensively adapted for the development of a diverse array of water-based OECTs featuring various conjugated polymers. The incorporation of these ethylene glycol side chains is believed to facilitate efficient electrolyte uptake, leading to enhanced stability of charge carriers, and ultimately promoting efficient charge storage and transport within aqueous media [19,20,21,22,23,24]. Regarding the results of these references, the findings have been summarized in Table S1 [19,21,25,26,27]. Nevertheless, it is worth noting that a significant portion of the existing literature relies on chemical synthesis to introduce ethylene glycol side chains, a time-consuming process that often uses palladium as a catalyst. However, the presence of residual excess palladium in the final product may deteriorate the overall device performance [28]. Hence, there is an urgent need to pursue rapid and general approaches to address this issue and advance the field of OECTs.

The rise in biological applications of OECTs has triggered the need for patterned circuits. Patterned fabrication processes enable the creation of repeated or continuous patterns using techniques such as inkjet printing [29,30,31,32], screen printing [33], roll-to-roll [34,35], and photolithography [36,37,38,39,40,41]. Among of them, photolithography utilizing optically triggered photo-crosslinkable reactions allows for the creation of patterned thin films with a resolution as fine as 10 µm [41]. Photo-crosslinkers containing at least two reactive groups, such as diazirines and azides, have been widely utilized in realizing crosslinked conjugated polymers through reactive carbene and nitrene insertion on nearby alkyl side chains of conjugated polymers under ultraviolet (UV) illumination, respectively [42,43,44,45,46,47].

Recently, several poly(ethylene glycol) (PEG) crosslinkers have been disclosed to enhance the performance of hydrophobic conjugated polymers in OECTs [48,49,50,51]. However, these PEG crosslinkers often require specialized, multi-step synthesis, which limits their accessibility and the widespread adoption of OECT-based integrated circuits for general biological applications.

Herein, commercially available PEG bisazide, a photo-crosslinking agent, is introduced into the p-type semiconducting polymers as active channels in accumulated-mode OECTs to investigate the effect of inserted hydrophilic ethylene glycol on the µC* and the response speed. Both hydrophobic conjugated polymers, namely poly{2,2′-[(2,5-bis(2-hexyldecyl)-3,6-dioxo-2,3,5,6-tetrahydropyrrolo[3,4-c]pyrrole-1,4-diyl)dithiophene]-5,5′-diyl-alt-thiophen-2,5-diyl}22 (DPP-3T) and poly{[2,3,5,6-tetrahydro-2,5-bis(2-octyldodecyl)-3,6-dioxopyrrolo[3,4-c]pyrrole-1,4-diyl]-2,5-thiophenediylthieno [3,2-b]thiophene-2,5-diyl-2,5-thiophenediyl} (DPP-DTT) demonstrate remarkable enhancement in the µC* and switch speed by one order of magnitude after photolithography, compared to pristine conjugated films. In addition, high resolution, as fine as 5 µm, and the depletion-mode inverter can be realized through the photolithography technique.

## 2. Materials and Methods

### 2.1. Materials

1,2-dichlorobenzene (anhydrous, 99.8%), chloroform (anhydrous, 99.8%), 1-ethyl-3-methylimidazolium bis(trifluoromethylsulfonyl)imide (EMIM TFSI) (HPLC, ≥98%), poly(vinylidene fluoride-co-hexafluoropropylene) (PVDF-HFP) (Mw ~ 400,000, Mn ~ 130,000, pellets), and poly(ethylene glycol) bisazide (azide) (Mn 20,000) were purchased from Sigma-Aldrich, St. Louis, MO, USA. Carbon nanotube (CNT) (single-walled 90% OD: <2 nm, L: <20 μm), poly{2,2′-[(2,5-bis(2-hexyldecyl)-3,6-dioxo-2,3,5,6-tetrahydropyrrolo [3,4-c]pyrrole-1,4-diyl)dithiophene]-5,5′-diyl-alt-thiophen-2,5-diyl} (PDPP-3T) (Mw > 30,000), and poly[[2,3,5,6-tetrahydro-2,5-bis(2-octyldodecyl)-3,6-dioxopyrrolo [3,4-c]pyrrole-1,4-diyl]-2,5-thiophenediylthieno [3,2-b]thiophene-2,5-diyl-2,5-thiophenediyl] (DPP-DTT) (Mw ~ 111,029) were purchased from Ossila, Sheffield, UK. Polydimethylsiloxane (PDMS), Sylgard-184, was purchased from Dow Chemical Company, Midland, MI, USA.

### 2.2. Fabrication of OECTs

The highly doped n-type silicon wafers with 300 nm thick SiO_2_ were cleaned by ultrasonic cleaner in toluene, acetone, and isopropyl alcohol before being used as substrates for OECTs. Bottom-contact and top-gate configuration of OECTs with a channel length (L) and width (W) of 50 μm and 1000 μm, respectively, was defined by 20 nm thick thermal evaporated chromium and 50 nm thick thermal evaporated gold as drain and source electrodes. Photo-crosslinkable active film with conjugated polymer-to-azide mass ratios of 1:0, 1:0.1, 1:0.5, 1:0.75, 1:1, 1:1.25, and 1:1.5 were prepared with a concentration of conjugated polymer fixed at 4 mg mL^−1^ for DPP-DTT and 1 mg mL^−1^ for DPP-3T. After the above solutions were spin-coated at 1500 rpm for 60 s, followed by 2000 rpm for 10 s on the above substrates, the active composite film was formed, then exposed to a UV lamp (254 nm, 1.47 mW cm^−2^) through a shadow mask for 30 min. After photo-crosslinking, the film was developed using chloroform at a spin rate of 4000 rpm for 10 s by a spin-coater to remove the non-crosslinked regions. All the above fabrication steps were conducted inside a glovebox. For the preparation of ion gel film, a solution was prepared with a mass ratio of 1:2:7 (PVDF-HFP:EMIM TFSI:acetone), stirred at room temperature to form a clear and uniform solution. The solution was spin-coated onto a 300 nm thick silicon dioxide substrate at 250 rpm/60 s. Afterward, the sample was placed on a 40 °C hot plate to remove acetone residue, yielding the ion gel, which was cooled at room temperature before use. Post-ion gel fabrication, a 0.5 mg ml^−1^ P3HT chloroform solution was prepared and stirred overnight at 60 °C. Subsequently, a CNT solution was created by dissolving 6 mg of CNT in 28 mL of chloroform and subjected to ultrasonic agitation for an hour, with the addition of 2 mL of 0.5 mg ml^−1^ P3HT chloroform solution to maintain suspension. The resulting CNT solution was spray-coated onto the ion gel to form a conductive film with a resistance <10 kΩ, serving as the top gate electrode. Finally, the ion gel/CNT was trimmed and attached to a substrate with a spin-coated polymer/azide composite thin film.

### 2.3. Fabrication of the Flexible OECT-Based NOT Logic Gate

Polydimethylsiloxane (PDMS) was utilized as an elastomeric adhesive layer to temporarily secure the flexible polyethylene naphthalate (PEN) film onto a rigid glass substrate during device fabrication. The two-part silicone elastomer kit (prepolymer base: curing agent = 20:1 by weight) was degassed, spin-coated, and thermally cured prior to PEN lamination. Inverters were realized by integrating a load transistor (*W*/*L* = 1000/100 µm) with a driver transistor of varying dimensions (*W*/*L* = 1000/100 µm or 100/100 µm). Under these geometric constraints, the corresponding conduction parameter ratios (*K*_D_/*K*_L_) were defined as 1 and 0.1, respectively. The 4 mg mL^−1^ DPP-DTT with polymer-to-azide ratio of 1:1 was spin-coated onto the PEN substrate with pre-deposited gold electrodes at 1500 rpm for 60 s, followed by 2000 rpm for 10 s.

A mask was placed over the coated substrate, and the sample was irradiated with UV light (254 nm) for 30 min to trigger photo-crosslinking reaction. The film was then developed by chloroform with a spin rate of 4000 rpm for 20 s. All the above fabrication steps were conducted inside a glovebox. Finally, the device was removed from the glovebox, and free-standing ion gel films were placed across the DPP-DTT-100 film and the side gate electrode.

### 2.4. Characterization

^1^H-nuclear magnetic resonance (NMR) spectra were recorded at 500 MHz, using a BRUKER spectrometer in DCB-d_4_. The chemical interaction between polymer and azide was analyzed using Fourier-transform infrared (FTIR, Miracle ATR, Perkin Elmer, Waltham, MA, USA). Transmission electron microscopy (TEM, JEM-2100F Electron Microscope, Tokyo, Japan) was used to investigate the morphology of composite films. The film retention was characterized using UV–Vis spectrum (UV–Vis-NIR Spectrophotometer, HITACHI U-4100, Tokyo, Japan). Atomic Force Microscopy (AFM, Bruker Dimension Icon Multi-Functional Scanning Probe Microscope, Santa Barbara, CA, USA) was used to measure the surface morphology images and average film thickness of both pure polymer films and composite films. A contact angle meter (4206 Contact Angle Meter, First Ten Angstroms, Portsmouth, VA, USA) was used to analyze the surface energy of composite films. The surface energy values of the films were calculated through the Girifalco–Good–Fowkes–Young (GGFY) equation:

(1) $$\left(1\;+\cos\theta \right)\gamma_{LV}\;=\;2\sqrt{\gamma_{SV}\gamma_{LV}}\;-\;\pi$$

where *θ* represents the contact angle, $\gamma_{LV}$ stands for the liquid–vapor interfacial energy, typically 72.8 mJm^−2^ at room temperature for water, and $\gamma_{SV}$ denotes the solid–vapor interfacial energy. *p* is the spreading pressure, which is often neglected. Grazing incident wide-angle X-ray scattering (GIWAXS), at Taiwan Photon Source (TPS) beamline 25A in the National Synchrotron Radiation Research Center (NSRRC), was executed to probe the crystalline domain of composite film. The film was probed with three-electrode-based electrochemical impedance spectroscopy (EIS) (PGSTAT204-Metrohm Autolab) to obtain capacitance at the frequency range between 10^−3^ and 10^6^ Hz with a set potential at 0 V and a sinusoidal signal with amplitude of 5 mV. The electrical characteristics of OECTs and flexible OECT-based NOT logic gates were probed by using Keithley 4200-SCS and Keithley 2636 semiconductor parameter analyzers.

### 2.5. Use of Generative AI for Graphic Illustration

**Figure 1:**
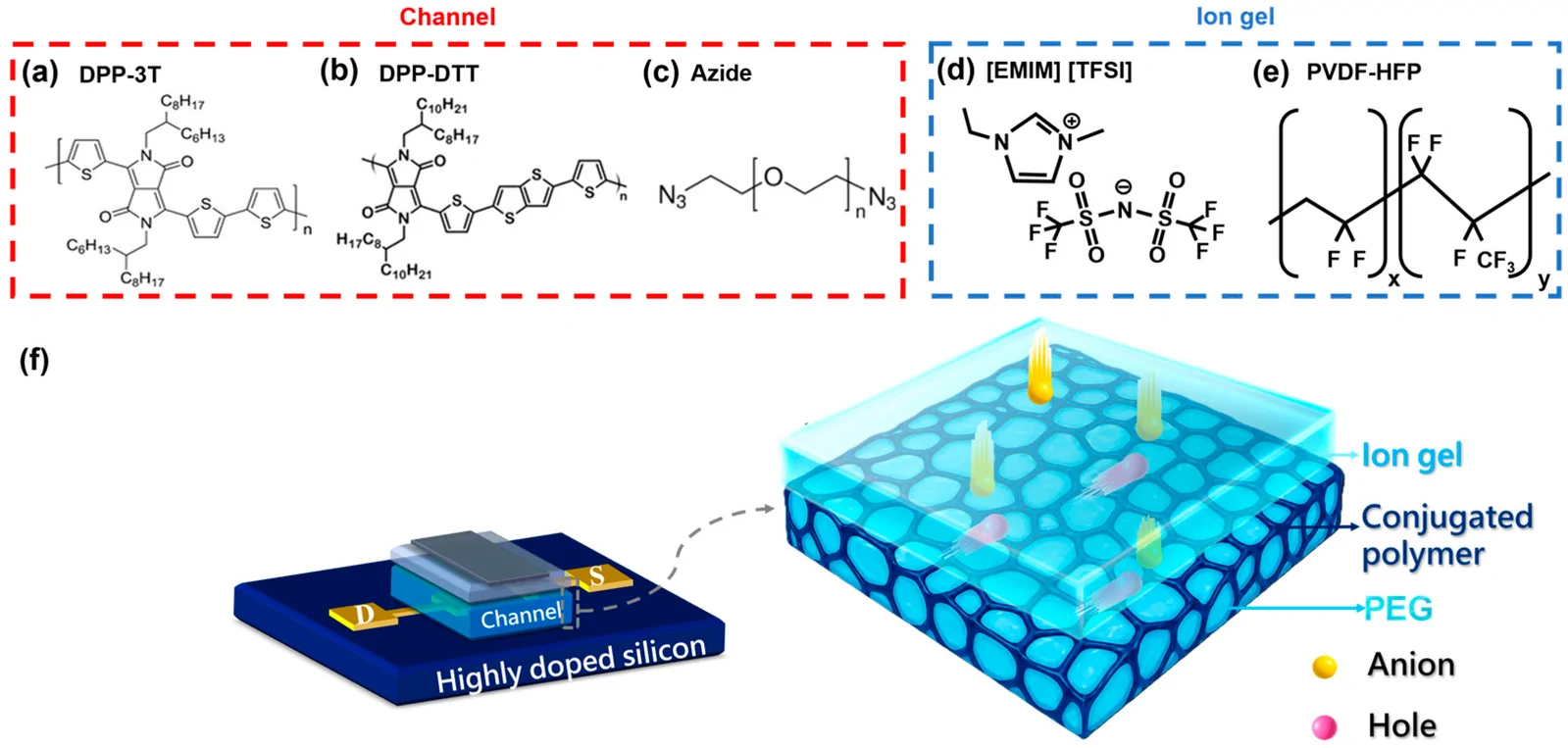
Chemical structures of (**a**) DPP-3T, (**b**) DPP-DTT, (**c**) azide, (**d**) [EMIM] [TFSI], (**e**) PVDF-HFP. (**f**) Schematic diagram with channel consisting of hydrophobic conjugated polymer and hydrophilic PEG. Hydrophobic conjugated polymer network is responsible for electronic charge transport. Hydrophilic PEG matrix provides dedicated pathways for volumetric electrolyte/ion transport. D and S denote the drain and source terminals, respectively.

A generative artificial intelligence tool, ChatGPT (GPT-5 model, OpenAI, San Francisco, CA, USA), was utilized to assist in rendering the 3D schematic representation of the OECT architecture and the associated thin-film surface morphology shown in Figure 1f. The experimental AFM topographic data obtained in this study was used as the primary reference. The specific prompt provided to the model was: *“Generate a 3D schematic illustration demonstrating the device configuration and film bicontinuous morphology for Figure 1f using the provided AFM image as a reference.”* All AI-generated graphical elements were subsequently reviewed, verified, and edited by the authors to ensure factual accuracy and consistency with experimental observations.

## 3. Results

### 3.1. Morphological Analysis of Photo-Patternable Composite Films

Figure 1a–e present the chemical structures adopted in the photo-patternable composite film-based OECTs. DPP-3T, and DPP-DTT were employed as the semiconducting polymers, with PEG bisazide (azide) as the photo-crosslinker for the preparation of photo-patternable composite films. Azide generates nitrene under UV light illumination and then joins two alkyl chains through nitrene insertion into C-H bonds, which enable crosslinked networks and antisolvent features for conjugated polymers with alkyl side chains (Figure S1a). To validate the proposed photo-crosslinking mechanism between the azide and the conjugated polymers, solution H1 nuclear magnetic resonance (H1 NMR) spectroscopy was performed on the DPP-DTT/azide blend in 1,2-dichlorobenzene-$d_{4}$ ($DCB\text{-}d_{4}$) both before and after UV exposure (254 nm, 1.47 mWcm^−2^, 30 min). As illustrated in Figure S1b, the proton signal characteristic of the azide methylene protons was assigned at 3.5 ppm. Following UV irradiation, the resonance absorption of the methylene protons on the alkyl chain centered at 1.68 ppm disappeared. Concurrently, the emergence of a broad peak at 2.13 ppm indicated the formation of a secondary amine. This spectral evolution confirms that the highly reactive nitrene intermediates successfully inserted into the C-H bonds along the side chains of the conjugated polymers.

Figure S2 depicts the fabrication process of the ion gel film and the conjugated polymer/azide composite film-based OECT. The photo-patternable composite films are named according to the mass ratio of the semiconducting polymer-to-azide content as follows: DPP-3T-0, DPP-3T-10, DPP-3T-50, DPP-3T-75, DPP-3T-100, DPP-3T-125, and DPP-3T-150 for DPP-3T-to-azide mass ratios of 1:0, 1:0.1, 1:0.5, 1:0.75, 1:1, 1:1.25, and 1:1.5, respectively; and DPP-DTT-0, DPP-DTT-10, DPP-DTT-50, DPP-DTT-75, DPP-DTT-100, DPP-DTT-125, and DPP-DTT-150 for DPP-DTT-to-azide mass ratios of 1:0, 1:0.1, 1:0.5, 1:0.75, 1:1, 1:1.25, and 1:1.5, respectively. All the composite films were illuminated by UV light (254 nm, 1.47 mWcm^−2^) with a dose of 2.65 J cm^−2^ for the photo-crosslinking reaction. Each sample was developed by chloroform, except DPP-3T-0 and DPP-DTT-0, for the following characterization and OECT fabrication. An ion gel consisting of PVDF-HFP and [EMIM][TFSI] was adopted as a solid-state electrolyte here. The schematic image of the OECT with bottom-contact and top-gate configuration is shown in Figure 1f, with a bulk heterojunction consisting of phase separation between hydrophobic conjugated polymer for electronic charge carriers and hydrophilic PEG segments for ionic charge carriers.

**Figure 2:**
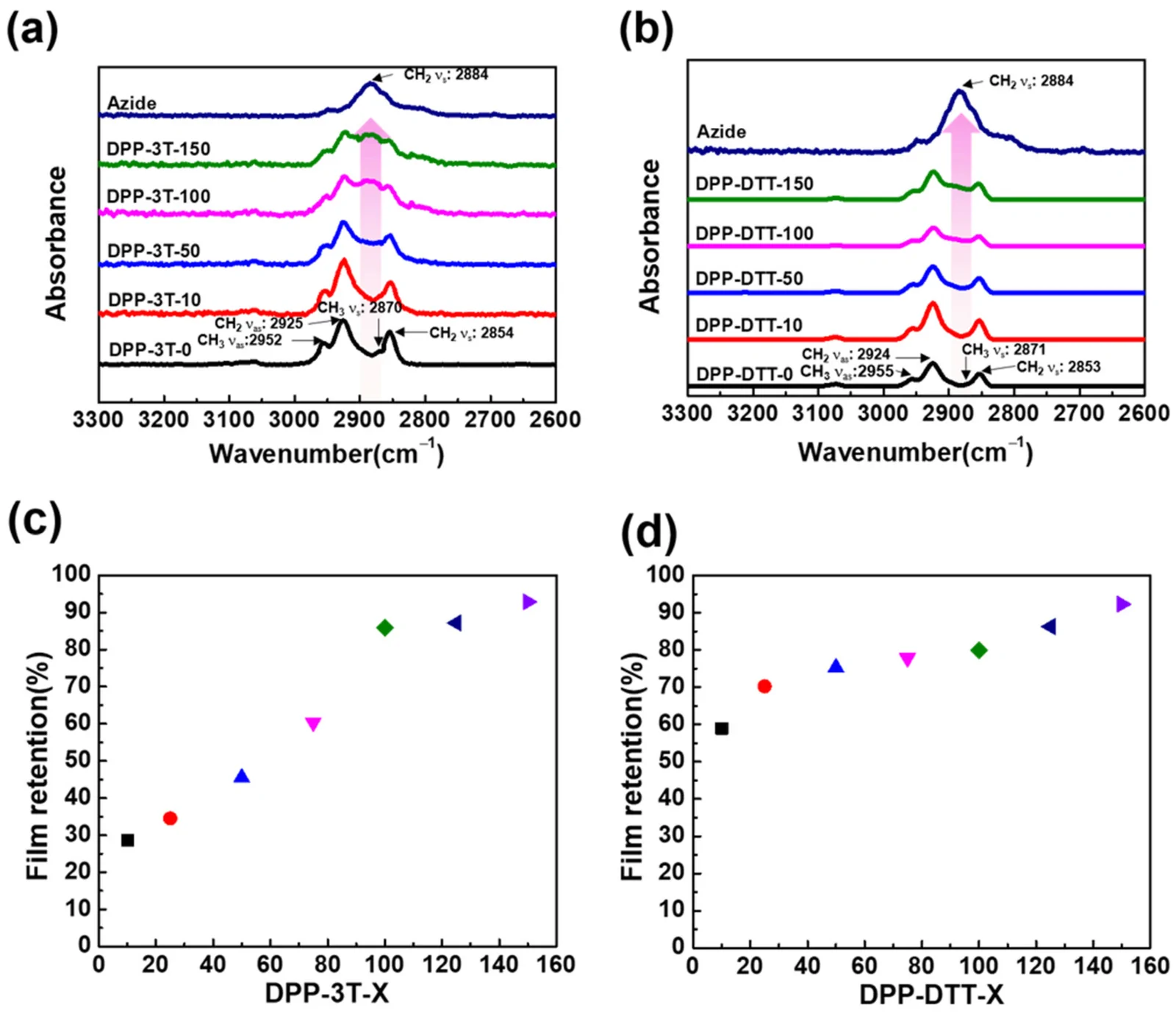
FTIR spectra of (**a**) DPP-3T/azide composite films and (**b**) DPP-DTT/azide composite films in the range of 2600 to 3300 cm^−1^. Film retention from UV–Vis spectra of (**c**) DPP-3T with different ratios of azide and (**d**) DPP-DTT with different ratios of azide.

Fourier-transform infrared spectroscopy (FTIR) was executed to probe the existence of PEG segments in the developed conjugated polymer/azide composite films. FTIR spectra in the range of 500 to 4000 cm^−1^ are demonstrated in Figure S3a,b for DPP-3T/azide and DPP-DTT/azide composite films, respectively. By zooming in to the localized region of 2600 to 3300 cm^−1^ (Figure 2a), the characteristic symmetric CH_2_ stretching signal at 2884 cm^−1^ assigned to PEG in pure azide can be observed. In addition, CH_2_ vibration (symmetric (ν_s_) at 2854 cm^–1^; asymmetric (ν_as_) at 2925 cm^–1^) and CH_3_ vibration (ν_s_ at 2870 cm^–1^ and ν_as_ at 2952 cm^–1^) belonging to the alkyl side chain of DPP-3T are retained in all the developed conjugated polymer/azide composite films, demonstrating that the photo-crosslinking by azide can be realized even with low azide content. The gradual increase in the intensity of the characteristic stretching signal of the CH_2_ at 2884 cm^−1^ with increasing azide content confirms the photoreaction of the azide group and the introduction of hydrophilic ethylene glycol into the composite films. Similar phenomena can be found in the DPP-DTT system, as shown in Figure 2b. With increasing azide content, the CH_2_ stretching signal at 2884 cm^−1^ can be distinguished eventually, and overlapped by the CH_2_ vibration and CH_3_ vibration of the alkyl side chain of DPP-DTT, demonstrating the universal strategy for the crosslinked network using azide as the photo-crosslinker.

To further quantify the composition in the composite film, ultraviolet–visible (UV–Vis) spectroscopy and grazing incident wide-angle X-ray scattering (GIWAXS) were executed. The extent of film retention can be determined by comparing the maximum absorbance of pre-developed and developed film at 837 nm for DPP-3T and 824 nm for DPP-DTT. As revealed in Figure S4, the decreasing absorbance difference with increasing azide content implies the photo-crosslinking reaction between azide and conjugated polymer. To quantify the residue of conjugated polymer in the developed film, the film retention is adopted and evaluated using the following formula:

(2) $$Film\;retention=\frac{Absorbance\;of\;the\;developed\;film}{Absorbance\;of\;the\;pre-developed\;film}\times 100\%$$

As demonstrated in Figure 2c,d, the plot of film retention against azide content reveals a positive correlation where retention reaches 93% for DPP-3T-150 and 92% for DPP-DTT-150. The high film retention in high azide content can be observed in GIWAXS as well. Figures S5 and S6 depict GIWAXS images of DPP-3T and DPP-DTT systems with varying polymer-to-azide mass ratios before and after development, respectively. Comparing in-plane 1D profiles between pre-developed films and developed films at different azide ratios reveals that higher azide ratios lead to better retention of crystalline signals in composite films. This trend is also observed in the scattering signals in the out-of-plane direction. No observable peak shifts suggest that the incorporated PEG segment is likely located either within the amorphous region of the conjugated polymer matrix or as phase-separated domains of PEG and the conjugated polymer.

**Figure 3:**
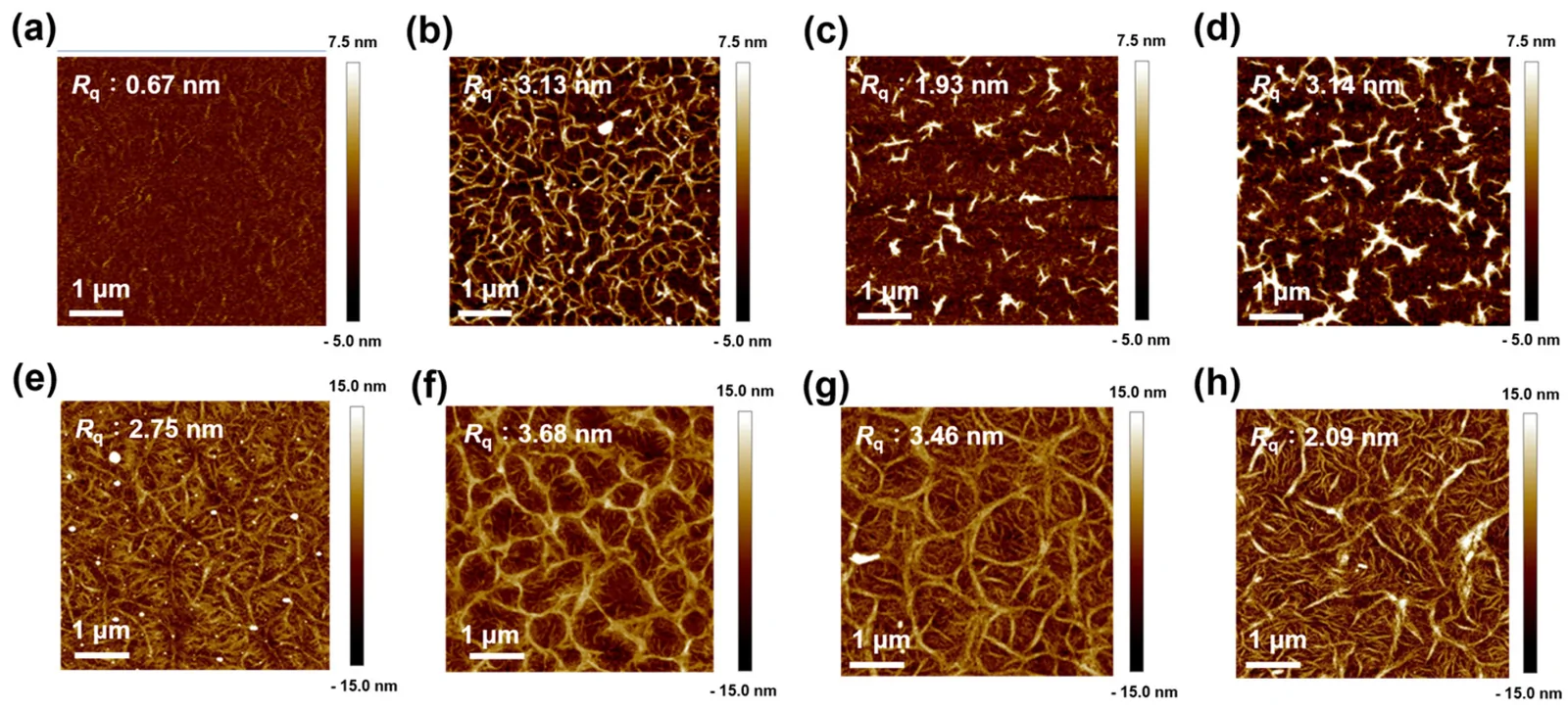
Morphology images of conjugated polymer/azide film with varying weight ratios of azide after development (**a**) DPP-3T-10, (**b**) DPP-3T-50, (**c**) DPP-3T-100, (**d**) DPP-3T-150, (**e**) DPP-DTT-10, (**f**) DPP-DTT-50, (**g**) DPP-DTT-100, and (**h**) DPP-DTT-150.

Atomic Force Microscopy (AFM) was utilized to reveal the surface morphology images of pure conjugated polymer and developed conjugated polymer/azide composite films. For the pure polymers, DPP-3T and DPP-DTT films exhibit needle-like crystalline morphologies (Figure S7a,b), whereas pure azide films display irregular and isolated aggregates (Figure S7c). In contrast, the DPP-3T/azide composite films progressively exhibit continuous network morphologies with sub-micrometer fibers (Figure 3a,b). With further increases in azide content, isolated branched fibers became predominant (Figure 3c,d). In the case of DPP-DTT/azide composite films, a denser network is observed as the azide content increases (Figure 3e–h). The observed morphological variations are likely attributed to phase separation between the fibrillar conjugated polymers and the PEG moiety. The resulting hydrophobic conjugated polymer network can provide lateral electronic charge transport, while leading to a maximized conjugated polymer/PEG interface.

**Figure 4:**
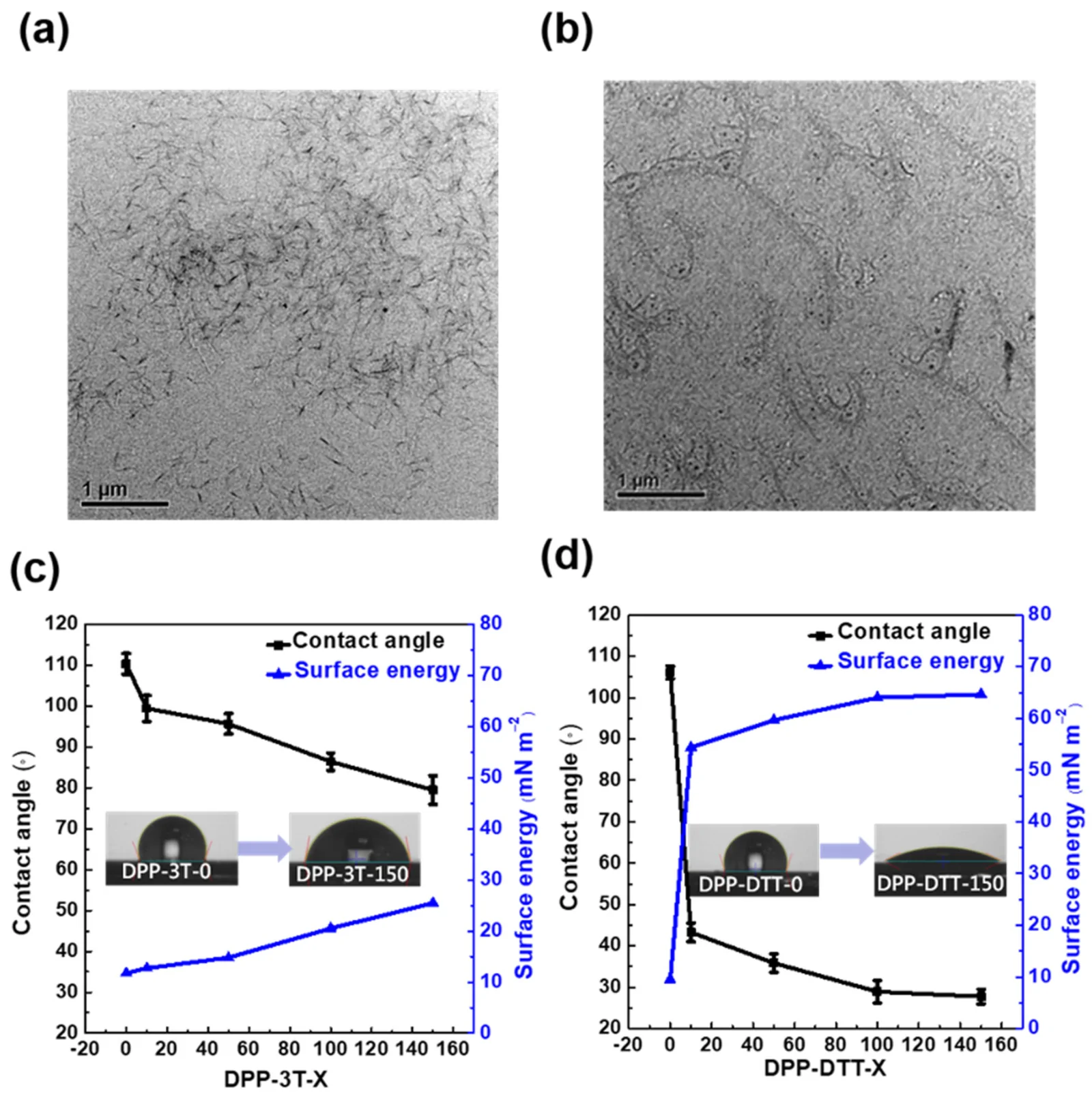
TEM images of (**a**) DPP-3T-50 and (**b**) DPP-DTT-100. Contact angle and surface energy of (**c**) DPP-3T series and (**d**) DPP-DTT series.

To clarify the spatial distribution of crystalline conjugated polymers, transmission electron microscopy (TEM) analysis was conducted. Due to the distinct contrast between the crystalline domains of the conjugated polymers and the PEG segments, the observed nanowires can be attributed to the crystalline domains of the conjugated polymers (Figure 4a,b). The homogeneous distribution of these nanowires indicates the potential for efficient lateral charge-carrier transport, even at elevated azide contents. Contact angle measurements were conducted on pure conjugated polymer and conjugated polymer/azide composite films using deionized water as the testing liquid. The images are shown in Figures S8 and S9, and the contact angles and surface energies obtained are summarized in Table S2. Figure 4c,d show the trend charts for DPP-3T/azide and DPP-DTT/azide composite films, respectively. The contact angles of DPP-3T-0 and DPP-DTT-0 films are 110 ± 3° and 106 ± 2°, respectively, both exceeding 90°, indicative of the high hydrophobicity characteristic of pristine conjugated polymers. Such hydrophobic surfaces generally inhibit ion penetration and limit interfacial electrolyte wetting. With increasing azide content, a monotonic decrease in contact angle accompanied by a corresponding increase in surface energy demonstrates that the wettability of the composite films can be effectively tuned by the incorporation of hydrophilic azide moieties. The markedly lower contact angle observed in the DPP-DTT system (28 ± 3° for DPP-DTT-150) compared with that of the DPP-3T system (79 ± 4° for DPP-3T-150). The phenomena may result from the highly crystalline or semi-crystalline polymers with strong π–π stacking (such as DPP-DTT) naturally inducing pronounced vertical segregation of PEG-azide crosslinkers, yielding rapid surface hydrophilization at lower azide ratios. In general, the incorporation of PEG via photo-crosslinking markedly increases surface hydrophilicity, which is expected to facilitate ion transport within the active layer and, consequently, to accelerate the device response in the resulting OECTs.

### 3.2. Characteristic and Performance of Conjugated Polymer/Azide Composite Film-Based OECTs and Depletion-Mode Inverter

**Figure 5:**
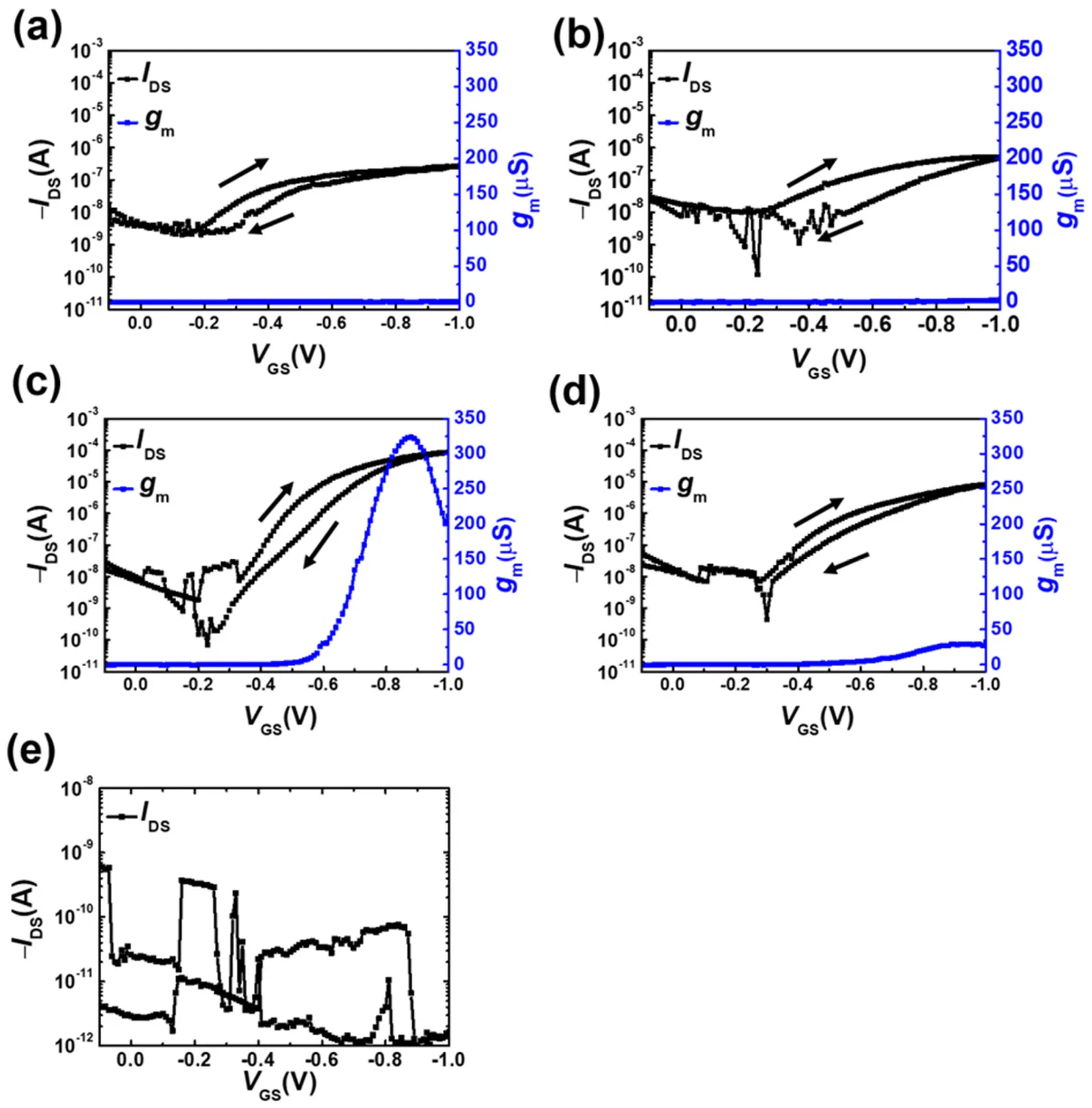
The transfer characteristics and *g*_m_-versus-*V*_GS_ curves of DPP-3T/azide-based OECTs: (**a**) DPP-3T-0, (**b**) DPP-3T-10, (**c**) DPP-3T-50, (**d**) DPP-3T-100, and (**e**) DPP-3T-150. *V*_GS_ was scanned with a step of 0.01 V and a delay of 1 s at a fixed *V*_DS_ of −1.0 V. The arrows in each subfigure indicate the scan direction.

**Figure 6:**
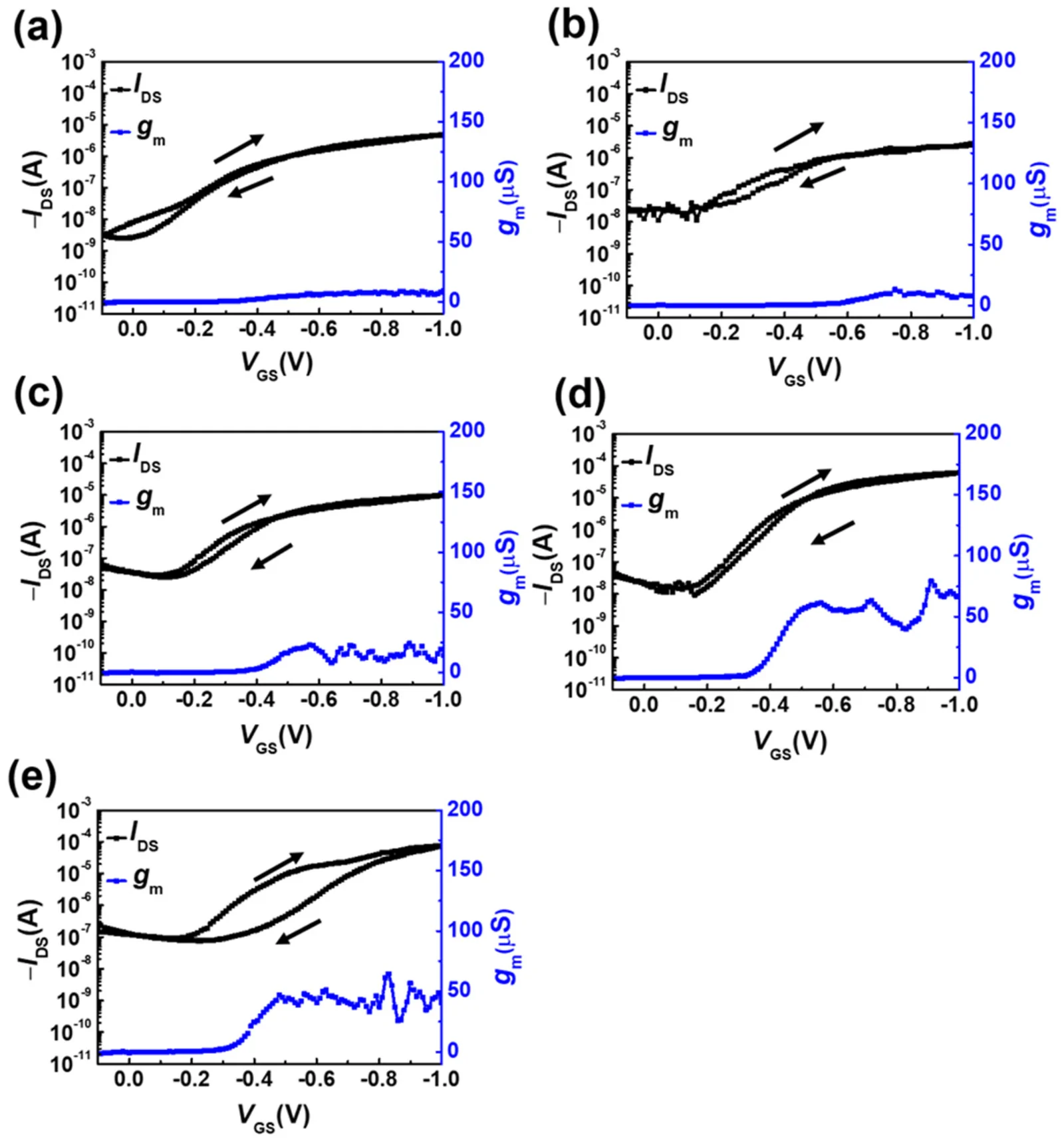
The transfer characteristics and *g*_m_-versus-*V*_GS_ curves of DPP-DTT/azide-based OECTs: (**a**) DPP-DTT-0, (**b**) DPP-DTT-10, (**c**) DPP-DTT-50, (**d**) DPP-DTT-100, and (**e**) DPP-DTT-150. *V*_GS_ was scanned with a step of 0.01 V and a delay of 1 s at a fixed *V*_DS_ of −1.0 V. The arrows in each subfigure indicate the scan direction.

To compare the performance of the conjugated polymer/azide composite film-based OECTs with different azide ratios, their transfer characteristics and output characteristics were probed. The transconductance, *g*_m_, is extracted (*g*_*m*_ = *∂I*_DS_/*∂V*_GS_ where *I*_DS_ is source-drain current and *V*_GS_ is gate-source voltage) and describes the extent to which an OECT can amplify a gate voltage signal (Figure 5 and Figure 6). Mobility-volumetric capacitance (μC*) product is extracted from the linear portion of *I*_DS_^1/2^-versus-*V*_GS_ curves according to the equation *I*_DS_^1/2^ = (*V*_TH_ − *V*_GS_) × ((*Wd*/2*L*) μC*) ^1/2^ (Figures S10 and S11), where *W*, *d*, and *L* are channel width, channel thickness, and channel length, respectively. In this study, the dimensions were set to *W* = 1000 µm and *L* = 50 µm. The channel thickness was probed by Atomic Force Microscopy (AFM) as demonstrated in Figure S12. In addition, to quantify the improvement in response speed, switching tests were conducted within the *V*_GS_ ranging from −0.5 V to −1.0 V (Figures S13 and S14). The probed *I*_DS_ was fitted by exponential curves for the rise and fall parts by Equations (3) and (4).

(3) IDS=A(1−e−tτON)

(4) IDS=A(e−tτOFF)

where *A* is the scaling factor and *τ*_ON_ and *τ*_OFF_ are the characteristic ON time and OFF time, respectively.

**Figure 7:**
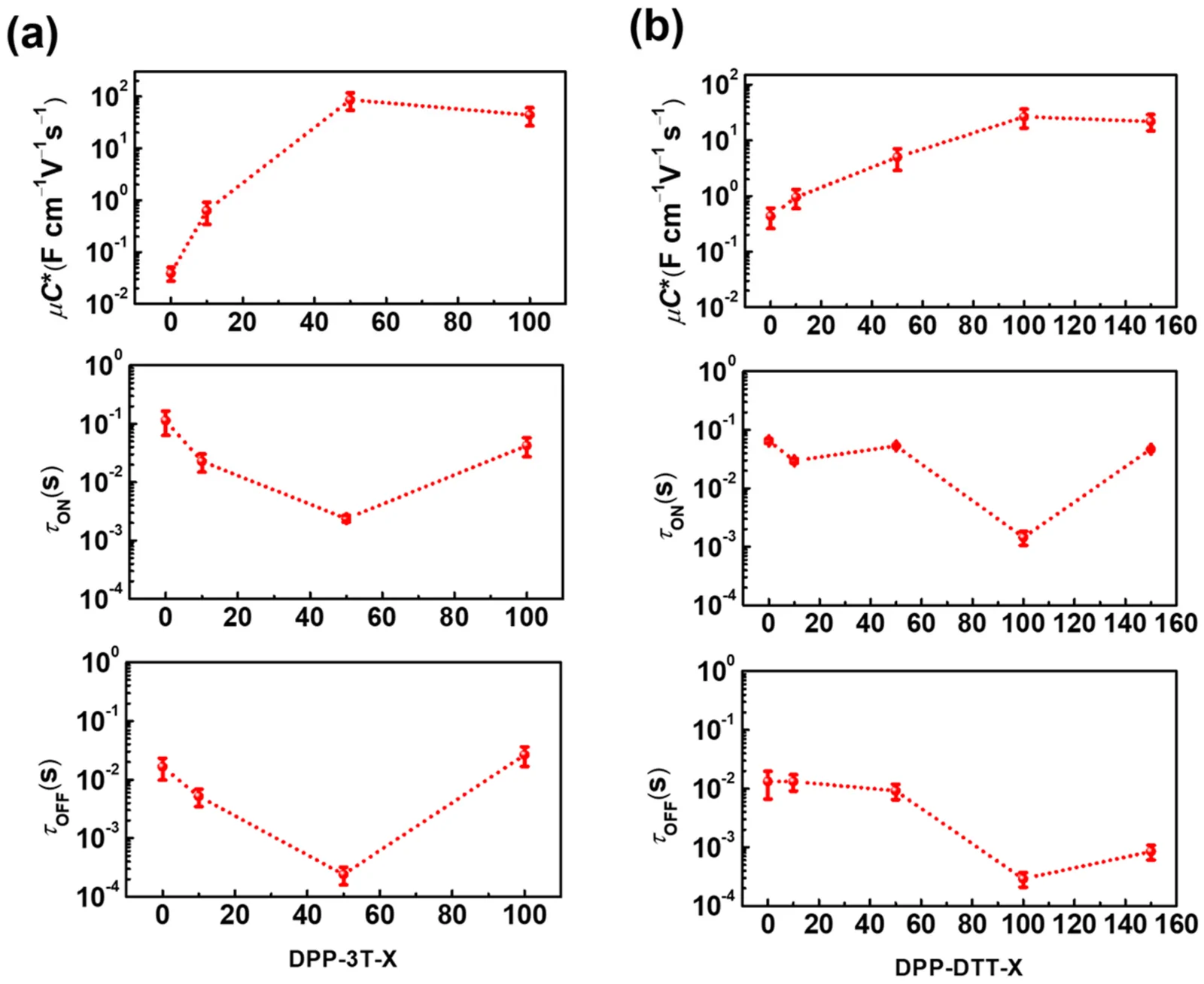
Overview of the electrical characteristics of conjugated polymer/azide-based OECTs: (**a**) DPP-3T/azide series and (**b**) DPP-DTT/azide series.

All the electrical properties are summarized in Tables S3 and S4. The maximum μC* product and minimum τ_ON_ and τ_OFF_ are observed at DPP-3T-50 and DPP-DTT-100 for DPP-3T and DPP-DTT series, respectively (Figure 7). It is interesting to note that the maximum μC* product and minimum τ_ON_ and τ_OFF_ occurred at same azide content, demonstrating both signal amplification and doping/de-doping dynamics rely on the bulk heterojunction formed by the hydrophilic and hydrophobic networks, as discussed above. However, an excessive amount of azide crosslinker in the composite film disrupts this optimized phase-separated morphology. As shown in Figure 3d, isolated branched fibrils are observed in DPP-3T-150, which likely impedes lateral electronic charge transport. Conversely, the overly dense network observed in DPP-DTT-150 may hinder vertical ionic penetration. While the ON current of DPP-DTT-150 is comparable to that of DPP-DTT-100, it displays observable transfer hysteresis. This behavior is attributed to excessive volume swelling and sluggish ion diffusion kinetics in the over-doped hydrophilic phase. Furthermore, compared to the DPP-3T system, the maximum μC* product does not fluctuate dramatically with the azide content in the DPP-DTT system, which accords with the uniform network for all DPP-DTT series (Figure 3e–h). The sub-millisecond-scale characteristic times for both optimized DPP-3T and DPP-DTT systems are suitable for biosensor applications and are comparable to those of the state-of-the-art patterned OECTs (Table S5) [40,52,53,54,55,56]. Our device demonstrates highly competitive performance compared to state-of-the-art solid-state OECTs, achieving a fast response time on the order of 10^−3^~10^−4^ s, a µC* product of 277 ± 155 F cm^−1^ V^−1^ s^−1^, and a high patterning resolution of 5 µm. Specifically, a recent study demonstrated a scalable approach by blending a commercial hydrophilic additive, poly(ethylene glycol) dimethacrylate (PEGDMA), into a hydrophobic semiconductor diketopyrrolopyrrole-3,4-ethylenedioxythiophene (DPP-EDOT), which improved the maximum *g*_m_ from 21.87 ± 2.57 mS to 53.42 ± 1.74 mS (a ~2-fold enhancement) [53]. In sharp contrast, our strategy utilizing the commercial PEG-bisazide photocrosslinker achieves a remarkable 355-fold enhancement (from 0.78 ± 0.53 µS to 277 ± 155 µS) in maximum *g*_m_ for the DPP-3T system. Therefore, we believe the proposed commercial PEG-bisazide crosslinker offers a highly competitive and facile solution for advancing photo-patternable, high-performance solid-state OECTs. The higher gate-modulated current in DPP-3T-50 and DPP-DTT-100 compared to pristine conjugated polymers in the output curve also confirms the formation of a hydrophilic pathway for electrolyte (Figure S15). The above results reveal that the incorporation of the hydrophilic photo-crosslinking agent in conjugated polymer is a universal and facile strategy for ionic and electronic charge carriers within the whole channel’s volume for OECTs, ensuring a reliable platform for integrated logic applications. To explore the existence of PEG-bisazide and its effect on the volumetric capacitance of the OECT, electrochemical impedance spectroscopy (EIS) experiments were conducted with the device configuration shown in Figure S16a,b, and the data obtained were analyzed by using an equivalent circuit with a resistor and a constant phase element (CPE) in series. In the Nyquist complex plane plot, an ideal capacitive response produces a straight line perpendicular to the real axis (an angle of 90° or π/2). However, for solid-state electrodes, the experimental impedance spectrum often displays a straight line with an angle slightly lower than π/2 due to microscopic surface roughness, interfacial inhomogeneities, and spatial capacitance dispersion. To accurately capture this non-ideal capacitive behavior, a constant phase element (CPE) in series with the solution resistance (*R*_s_) was employed for equivalent circuit fitting, where its impedance is expressed as:

(5) $${\hat{Z}}_{\mathrm{CPE}}\;=\;\frac{1}{T{\left(j\omega \right)}^{\phi }}$$

In our fitted results, the exponential factor ϕ was found to be around 0.8 for all the devices, which is very close to 1. Because ϕ ≈ 1, the CPE component behaves predominantly as an ideal capacitor. Therefore, the parameter *T* was directly used as the effective capacitance value to calculate the volumetric capacitance (C*) by dividing *T* by the channel film volume (Area (*A*) × Thickness (*d*)):

(6) $$C*\;=\;\frac{T}{A\;\times \;d}$$

Figure S16c,d show the Nyquist plots of DPP-3T and DPP-DTT systems, respectively. According to the fitting results, the *T* values of DPP-3T-0, DPP-3T-50, DPP-DTT-0, and DPP-DTT-100 are (1.88 ± 0.79) × 10^−5^ F, (3.28 ± 1.58) × 10^−5^ F, (1.80 ± 0.43) × 10^−5^ F, and (2.55 ± 0.48) × 10^−5^ F, respectively. The *A* is 0.125 cm^2^ for all devices, and the *d* of DPP-3T-0, DPP-3T-50, DPP-DTT-0, and DPP-DTT-100 are 3.56 ± 0.02 nm, 5.64 ± 0.24 nm, 16.40 ± 0.10 nm, and 11.33 ± 0.21 nm, respectively. Based on Equation (4), the calculated volumetric capacitances are 422 ± 178 F cm^−3^, 465 ± 224 F cm^−3^, 88 ± 21 F cm^−3^, and 180 ± 34 F cm^−3^ for DPP-3T-0, DPP-3T-50, DPP-DTT-0, and DPP-DTT-100, respectively. The carrier mobility (μ) was decoupled and calculated from μC* using the C* obtained from EIS ($\mu \;=\;\frac{\mu C*}{C*}$). The corresponding *m* can be obtained using µC* divided by C*. The calculated mobility values are (9.29 ± 4.80) × 10^−5^ cm^2^ V^−1^ s^−1^, (1.84 ± 1.12) × 10^−1^ cm^2^ V^−1^ s^−1^, (4.94 ± 2.30) × 10^−3^ cm^2^ V^−1^ s^−1^, and (1.48 ± 0.57) × 10^−1^ cm^2^ V^−1^ s^−1^ for DPP-3T-0, DPP-3T-50, DPP-DTT-0, and DPP-DTT-100, respectively. As summarized in Tables S3 and S4, the hydrophilic azide-modified conjugated polymers show higher volumetric capacitances than those of the pure ones, implying that a substantially larger amount of ionic liquid is involved in the doping process after the introduction of hydrophilic PEG in the channel. The substantially enhanced hole mobility observed in the composite systems compared to the pristine conjugated polymers is consistent with the GIWAXS results, which reveal enhanced (010) π–π stacking for DPP-3T composites and intensified (100) lamellar ordering for DPP-DTT composites.

**Figure 8:**
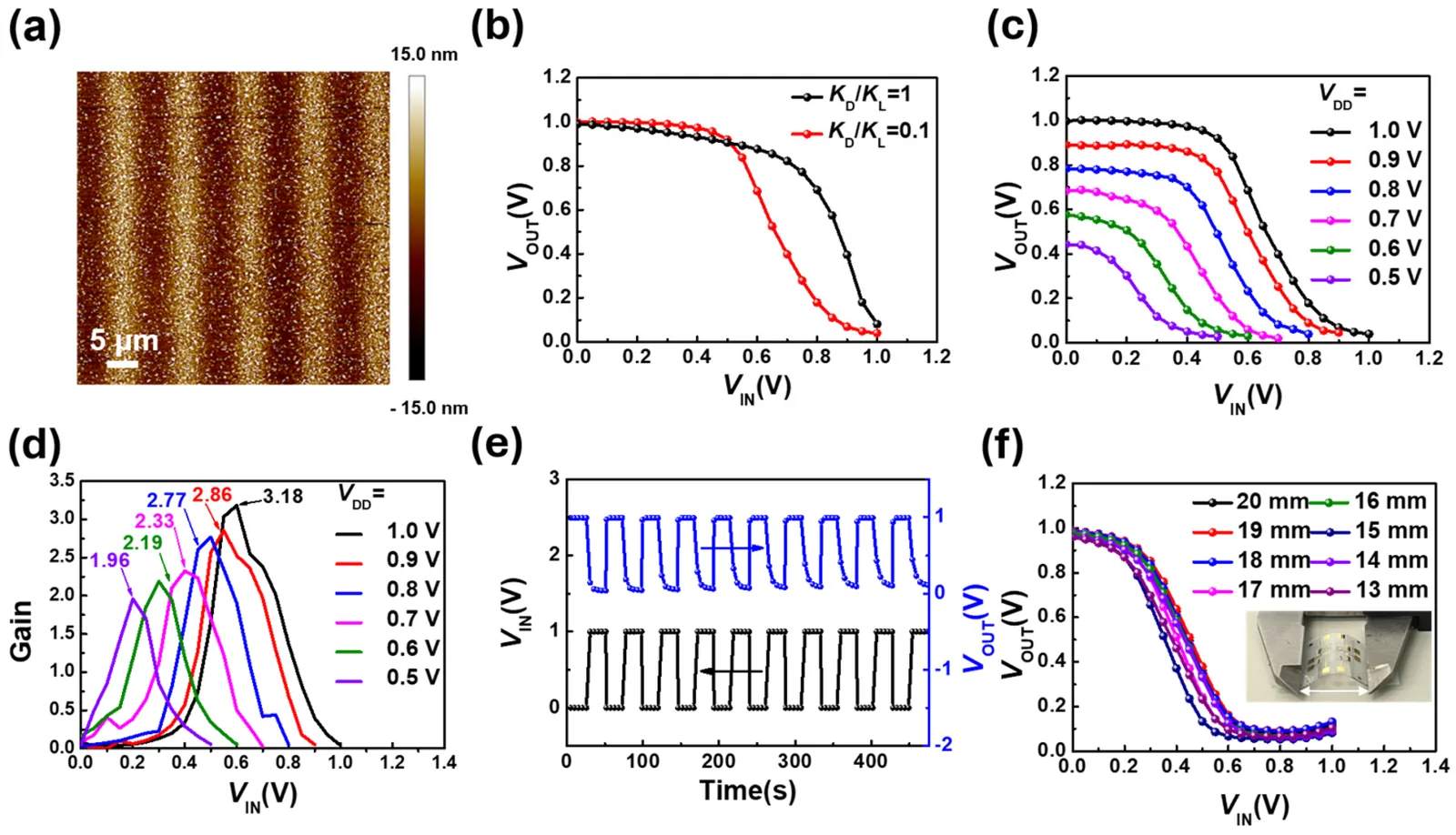
(**a**) AFM height image of photo-patterned DPP-DTT-100 film with line gap of 5 µm. (**b**) Voltage transfer curves of depletion-mode inverters with *K*_D_/*K*_L_ of 0.1 and 1, respectively. (**c**) Voltage transfer curves and (**d**) the gain values under various power supplies ranging from 1.0 V to 0.5 V. (**e**) Time traces of the input and the corresponding output voltage. (**f**) Voltage transfer curves under various chord lengths (the length of the double arrow line) ranging from 20 mm to 13 mm with *V*_DD_ = 1.

Leveraging this patternable characteristic, bespoke masks were adopted for resolution testing and the fabrication of a flexible NOT logic gate. Under the same UV illumination dose, the DPP-DTT-100 film was successfully photo-patterned with features down to 5 µm (Figure 8a). Taking advantage of the side-gate structure enabled by the OECT platform, depletion-load p-type OECT was employed to create a flexible OECT-based NOT logic gate. The detailed fabrication steps for the flexible OECT-based NOT logic gate are shown in Figure S17a–d. The conduction parameter ratios (*K*_D_/*K*_L_) for the driver and load devices are 0.1 and 1, respectively. With *V*_DD_ = 1 V, sweeping *V*_IN_ from 0 V to 1 V results in the reduction of *V*_OUT_ from 1 V to 0 V, clearly demonstrating the inverter operation (Figure 8b). The steeper voltage transfer curve can be observed in the NOT logic gate with *K*_D_/*K*_L_ of 0.1. Progressive reduction of *V*_DD_ lowers the transfer-curve slope and gain, attributable to the concomitant reduction in *V*_GS_ and the associated channel current (Figure 8c,d). In time-domain measurements at *V*_DD_ = 1 V, toggling *V*_IN_ between 0 V and 1 V yields rapid switching of *V*_OUT_ between 1 V and 0 V (Figure 8e), thereby verifying that the photo-crosslinked channel can be employed to complex circuit design. The flexible OECT inverter also retains stable transfer characteristics under bending at chord lengths ranging from 20 mm to 13 mm, confirming the device’s excellent stability under mechanical deformation (Figure 8f).

## 4. Discussion

The bulk heterojunction between hydrophilic ethylene glycol moieties and hydrophobic conjugated polymers can be formed by the insertion of nitrogen from photo-generated nitrene of azide into C-H bonds in the conjugated polymers. By precisely tuning the azide-to-polymer mass ratio, morphology can be optimized to improve ion injection and lateral electronic transport, yielding a characteristic ON-response time reduced by over one order of magnitude relative to pristine polymers. Leveraging the photo-patternability of the process, flexible OECT-based NOT logic gates are readily fabricated and operate at low supply voltages, down to 0.5 V. This facile and generalizable approach for conventional conjugated polymers expands the design space of accumulation-mode OECTs for bioelectronic applications.

## Data Availability

The data supporting this article have been included as part of the Supplementary Information.

## Supplementary Materials

The following supporting information can be downloaded at: https://www.mdpi.com/article/10.3390/polym18172057/s1, Figure S1: (a) Photo-crosslinking mechanism. (b) ^1^H NMR spectra of DPP-DTT/azide solution in DCB-d4 before and after UV light exposure; Figure S2: Fabrication process of conjugated polymer/azide composite film-based OECT; Figure S3: FTIR spectra of (a) DPP-3T/azide composite films and (b) DPP-DTT/azide composite films; Figure S4: UV–Vis results of DPP-3T with varying weight ratios of azide before and after development. (a) DPP-3T-10, (b) DPP-3T-50, (c) DPP-3T-100, and (d) DPP-3T-150. UV–Vis results of DPP-DTT with varying weight ratios of azide before and after development. (f) DPP-DTT-10, (g) DPP-DTT-50, (h) DPP-DTT-100, and (i) DPP-DTT-150; Figure S5: Comparison of in-plane and out-of-plane GIWAXS 1D patterns of DPP-3T with varying weight ratios of azide before and after development. In-plane direction analysis of (a) DPP-3T-10, (b) DPP-3T-50, (c) DPP-3T-100, and (d) DPP-3T-150. Out-of-plane direction analysis of (e) DPP-3T-10, (f) DPP-3T-50, (g) DPP-3T-100, and (h) DPP-3T-150; Figure S6: Comparison of in-plane and out-of-plane GIWAXS 1D patterns of DPP-DTT with varying weight ratios of azide before and after development. In-plane direction analysis of (a) DPP-DTT-10, (b) DPP-DTT-50, (c) DPP-DTT-100, and (d) DPP-DTT-150. Out-of-plane direction analysis of (e) DPP-DTT-10, (f) DPP-DTT-50, (g) DPP-DTT-100, and (h) DPP-DTT-150; Figure S7: Morphology images of pure polymers. (a) pure DPP-3T, (b) pure DPP-DTT, and (c) pure azide; Figure S8: Contact angle images of DPP-3T with varying weight ratios of azide after development. (a) DPP-3T-0, (b) DPP-3T-10, (c) DPP-3T-50, (d) DPP-3T-100, and (e) DPP-3T-150; Figure S9: Contact angle images of DPP-DTT with varying weight ratios of azide after development. (a) DPP-DTT-0, (b) DPP-DTT-10, (c) DPP-DTT-50, and (d) DPP-DTT-100, (e) DPP-DTT-150; Figure S10: Transfer curve and *I*_DS_^1/2^ and *V*_GS_ curves of representative OECTs based on (a) DPP-3T-0, (b) DPP-3T-10, (c) DPP-3T-50, and (d) DPP-3T-100. *V*_GS_ were scanned with step of 0.01 V and delay of 1 s. *μC** product is extracted from the linear portion of *I*_DS_^1/2^-versus-*V*_GS_ curves according to the equation *I*_DS_^1/2^ = (*V*_TH_ − *V*_GS_) × ((Wd/2L) *μC**) ^1/2^; Figure S11: Transfer curve and *I*_DS_^1/2^ and *V*_GS_ curve of representative OECT based on (a) DPP-DTT-0, (b) DPP-DTT-10, (c) DPP-DTT-50, (d) DPP-DTT-100, and (e) DPP-DTT-150. *V*_GS_ were scanned with step of 0.01 V and delay of 1 s. *μC** product extracted from the linear portion of *I*_DS_^1/2^-versus-*V*_GS_ curves according to the equation *I*_DS_^1/2^ = (*V*_TH_ − *V*_GS_) × ((Wd/2L) *μC**) ^1/2^; Figure S12: One-dimensional profile from height mode AFM. (a) DPP-3T-0, (b) DPP-3T-10, (c) DPP-3T-50, (d) DPP-3T-100, (e) DPP-3T-150, (f) DPP-DTT-0, (g) DPP-DTT-10, (h) DPP-DTT-50, (i) DPP-DTT-100, and (j) DPP-DTT-150; Figure S13: Response time testing of conjugated polymer/azide composite film-based (a) DPP-3T-0, (b) DPP-3T-10, (c) DPP-3T-50, and (d) DPP-3T-100. *V*_DS_ was fixed at −1 V while *V*_GS_ was switched from −0.5 V to −1.0 V; Figure S14: Response time testing of conjugated polymer/azide composite film-based (a) DPP-DTT-0, (b) DPP-DTT-10, (c) DPP-DTT-50, (d) DPP-DTT-100, and (e) DPP-DTT-150. *V*_DS_ was fixed at −1 V while *V*_GS_ was switched from −0.5 V to −1.0 V; Figure S15: Output characteristics of (a) DPP-3T-0, (b) DPP-3T-50, (c) DPP-DTT-0, and (d) DPP-DTT-100. Sweep delay time: 1 s; Figure S16: Electrochemical impedance spectroscopy (EIS) element (a) schematic diagram and simulated equivalent circuit and (b) top view with red-dotted rectangle demonstrating effective area (0.125 cm^2^). Comparison of Nyquist plots of conjugated polymers before and after adding the optimal weight ratio of azide: (c) DPP-3T-0 and DPP-3T-50, (d) pure DPP-DTT-0 and DPP-DTT-100; Figure S17: (a) Fabrication process of flexible OECT-based NOT logic gate. (b) The mask for Cr and Au electrodes with *K*_D_/*K*_L_ = 0.1 and *K*_D_/*K*_L_ = 1. (c) The mask for patterned conjugated polymer channel. (d) Image of the flexible OECT-based NOT logic gate; Table S1: A summarized list of chemical structure modification for improving the response speed of OECTs; Table S2: Summary of contact angle analysis results; Table S3: Overview of the OECT characteristics based on DPP-3T series; Table S4: Overview of the OECT characteristics based on DPP-DTT series; Table S5: Summary of electrical characteristics for state-of-the-art patterned OECTs.
