# Supplementary material for: Photo-Patternable Organic Electrochemical Transistors with Hydrophilic and Hydrophobic Bulk Heterojunction Enabled by Ethylene Glycol-Based Photo-Crosslinker
Source: Polymers (Basel). 2026 Aug 25;18(17):2057. doi: 10.3390/polym18172057 (PMC13568013; doi:10.3390/polym18172057)
Supplement: Supplementary file 1 [file polymers-18-02057-s001.zip › polymers-4491013-supplementary.pdf]

Photo-Patternable Organic Electrochemical Transistors with Hydrophilic and Hydrophobic Bulk Heterojunction Enabled by Ethylene Glycol-Based Photo-Crosslinker

Gu-Hao Cai [1,†], Yun-Cheng Guo [1,†], Sin-Rong Huang [1], Po-Hsiang Fang [1] and Jung-Yao Chen [1,2,3,*]

1 Department of Photonics, National Cheng Kung University, Tainan 70101, Taiwan
2 Academy of Innovative Semiconductor and Sustainable Manufacturing, National Cheng Kung University, Tainan 70101, Taiwan
3 Meta-nano Photonics Center, National Cheng Kung University, Tainan 70101, Taiwan
* Correspondence: jyc@gs.ncku.edu.tw
† These authors contributed equally to this work.

Table S1. A summarized list of chemical structure modification for improving the response speed of OECTs.

Active layer	Gate dielectrics	g_m (mS)	C^* (F cm^{-3})	μC^* (F cm^{-1} V^{-1} s^{-1})	μ (cm^2 V^{-1} s^{-1})	τ_{ON} (s)	τ_{OFF} (s)	Ref.
p(g2T-TT)	0.1 M NaCl	5.40	240	304.8	1.27	4.2×10^{-4}	4.3×10^{-5}	*Proc. Natl. Acad. Sci. U.S.A.* 2016, *113*, 12017. [1]
g-100%	0.1 M NaCl	18.8	297	N/A	0.55	1.16×10^{-4}	N/A	*Adv. Funct. Mater. 2020, 30, 1907657. [2]*
P3MEEMT	100 mM KCl	N/A	215.5	49.1 ± 5.0	0.23	8.2×10^{-1}	6.3×10^{-1}	*J. Mater. Chem. A* 2022, *10*, 10738.[3]
p(C8-T2)	0.1 M NaCl	0.15	342	0.13	$3.76 * 10^{-4}$	1.27×10^{-2}	N/A	*ACS Appl. Mat. Interfaces* 2021, *13*, 4253.[4]
gDPP-g2T	PBS	384.1 ± 17.8	N/A	N/A	N/A	4.25×10^{-4}	8.5×10^{-5}	*Nature* 2023, 613, 496. [5]
Homo-gDPP	PBS	251.2 ± 7.6	N/A	N/A	N/A	3.66×10^{-4}	4.5×10^{-5}	*Nature* 2023, 613, 496. [5]

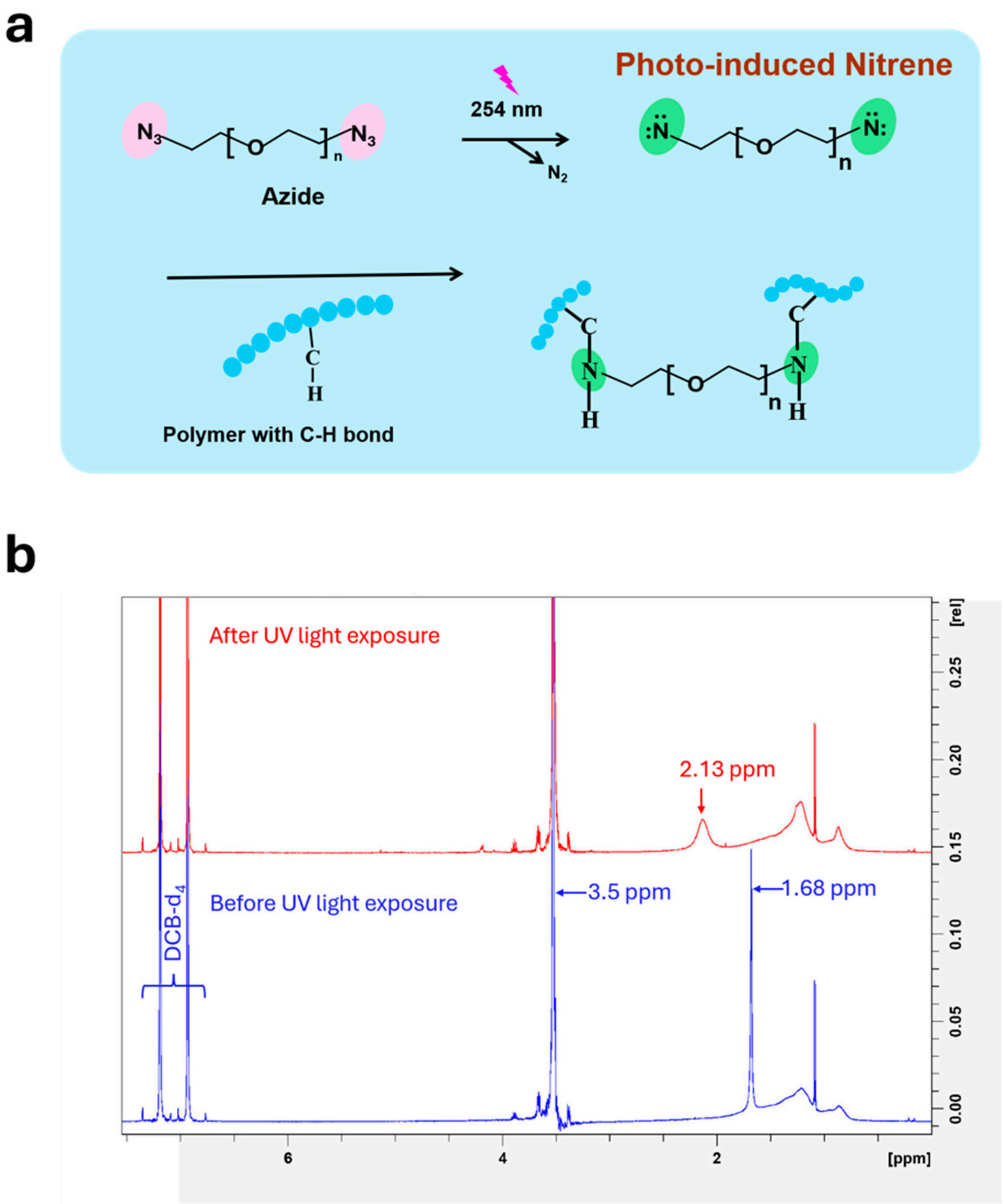

Figure S1. (a) Photo-crosslinking mechanism. (b)^{1}H NMR spectra of DPP-DTT/azide solution in $DCB-d_4$ before and after UV light exposure.

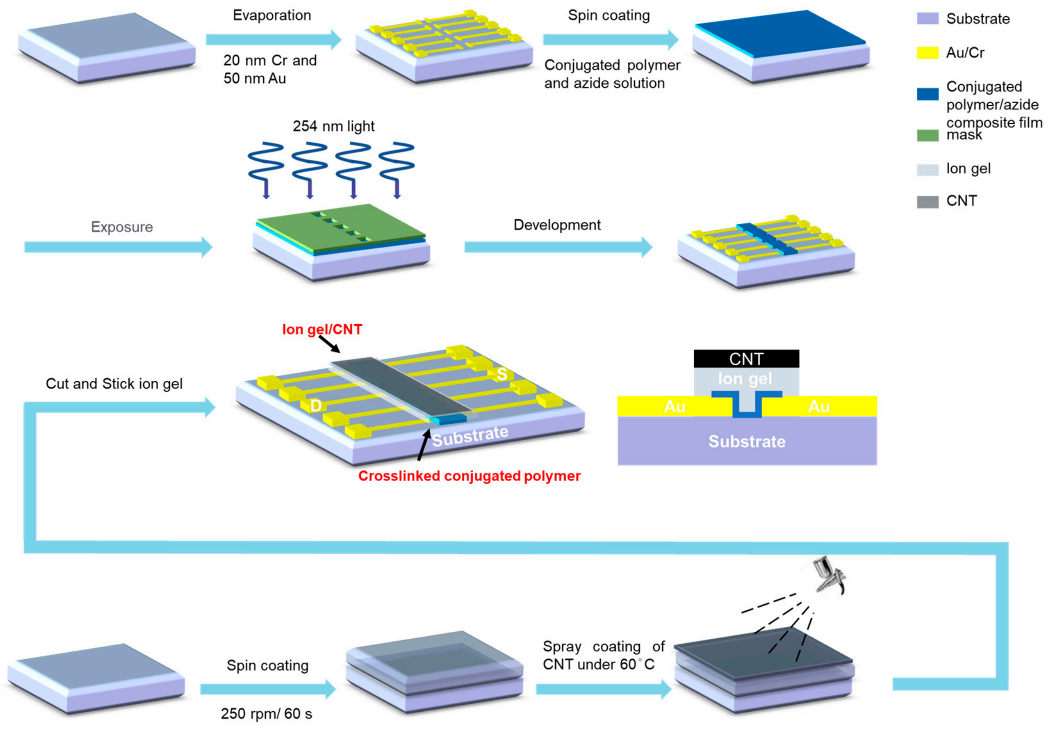

Figure S2. Fabrication process of conjugated polymer /azide composite film-based OECT.

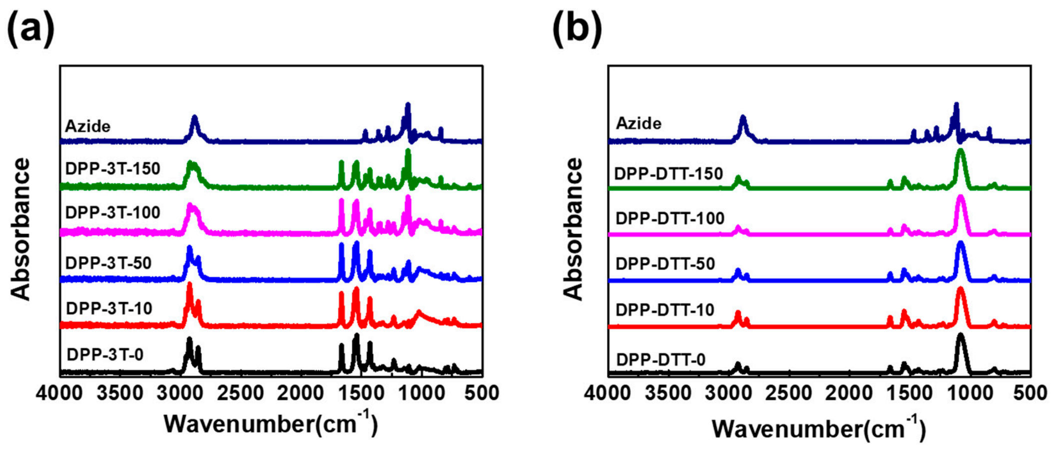

Figure S3. FTIR spectra of (a) DPP-3T/azide composite films and (b) DPP-DTT/azide composite films.

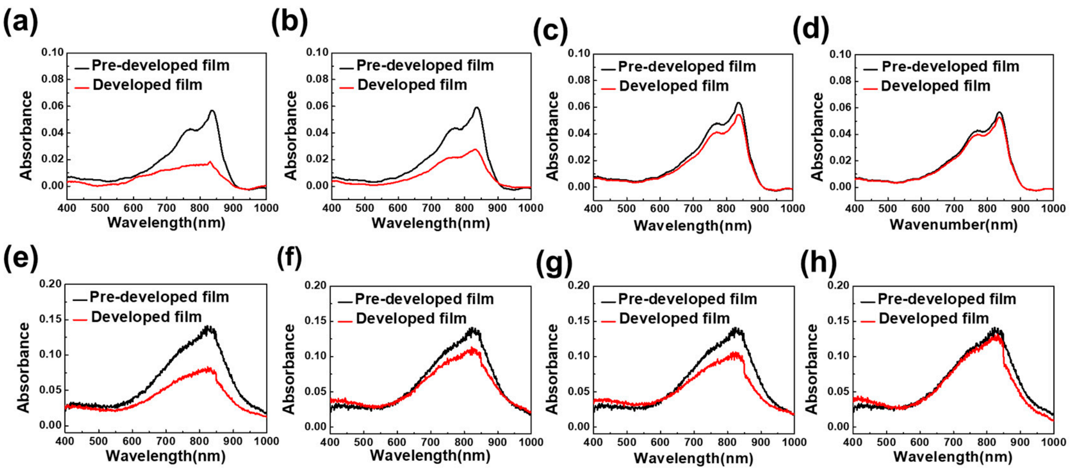

Figure S4. UV–Vis results of DPP-3T with varying weight ratios of azide before and after development. (a) DPP-3T-10, (b) DPP-3T-50, (c) DPP-3T-100, and (d) DPP-3T-150. UV–Vis results of DPP-DTT with varying weight ratios of azide before and after development. (f) DPP-DTT-10, (g) DPP-DTT-50, (h) DPP-DTT-100, and (i) DPP-DTT-150.

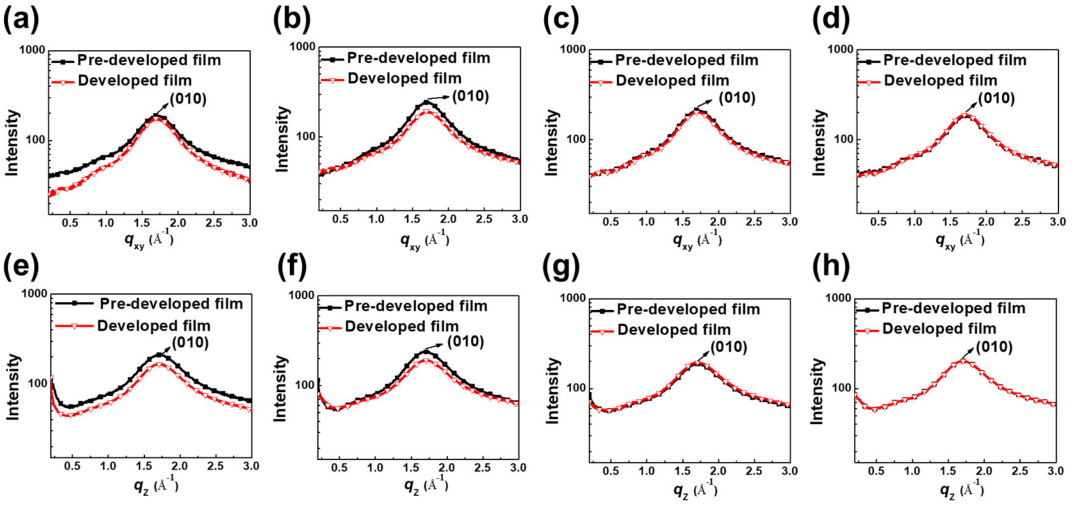

Figure S5. Comparison of in-plane and out-of-plane GIWAXS 1D patterns of DPP-3T with varying weight ratios of azide before and after development. In-plane direction analysis of (a) DPP-3T-10, (b) DPP-3T-50, (c) DPP-3T-100, and (d) DPP-3T-150. Out-of-plane direction analysis of (e) DPP-3T-10, (f) DPP-3T-50, (g) DPP-3T-100, and (h) DPP-3T-150.

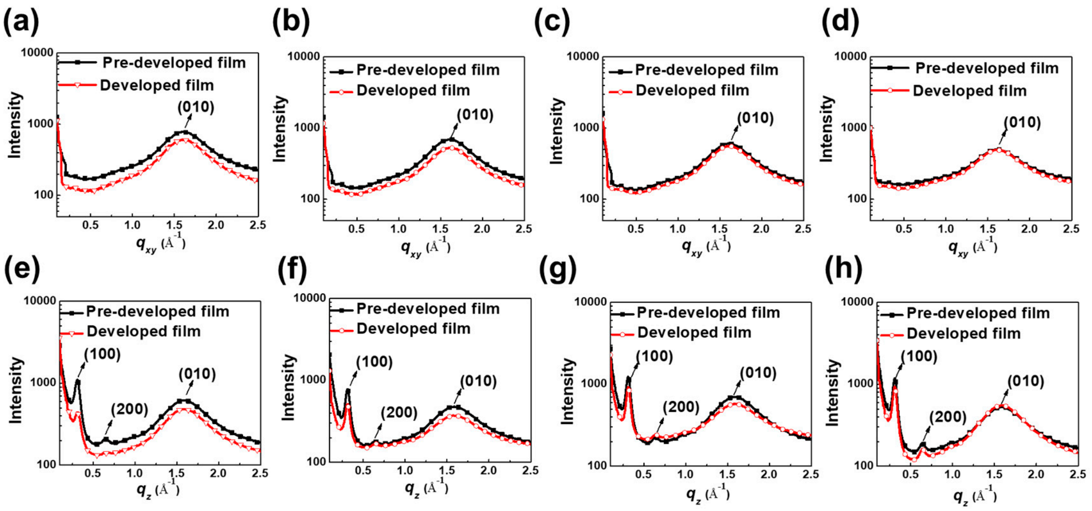

Figure S6. Comparison of in-plane and out-of-plane GIWAXS 1D patterns of DPP-DTT with varying weight ratios of azide before and after development. In-plane direction analysis of (a) DPP-DTT-10, (b) DPP-DTT-50, (c) DPP-DTT-100, and (d) DPP-DTT-150. Out-of-plane direction analysis of (e) DPP-DTT-10, (f) DPP-DTT-50, (g) DPP-DTT-100, and (h) DPP-DTT-150.

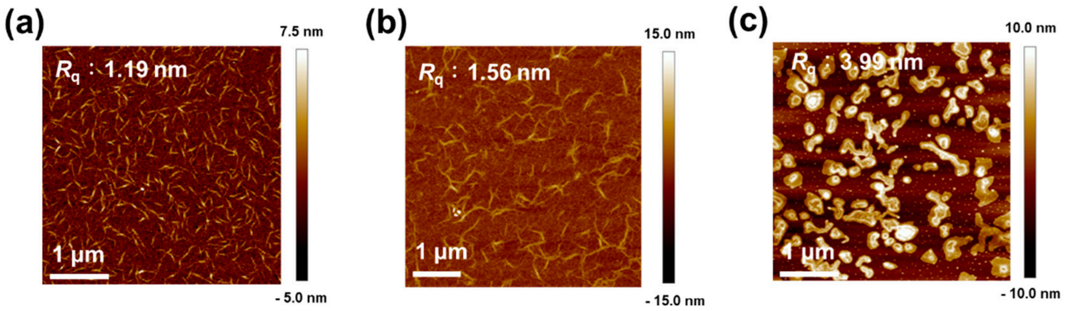

Figure S7. Morphology images of pure polymers. (a) pure DPP-3T, (b) pure DPP-DTT, and (c) pure azide.

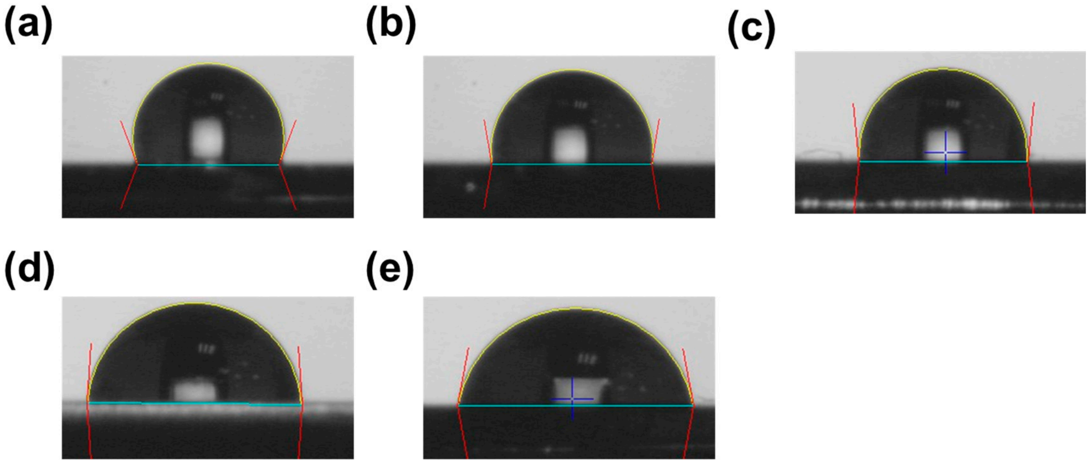

Figure S8. Contact angle images of DPP-3T with varying weight ratios of azide after development. (a) DPP-3T-0, (b) DPP-3T-10, (c) DPP-3T-50, (d) DPP-3T-100, and (e) DPP-3T-150.

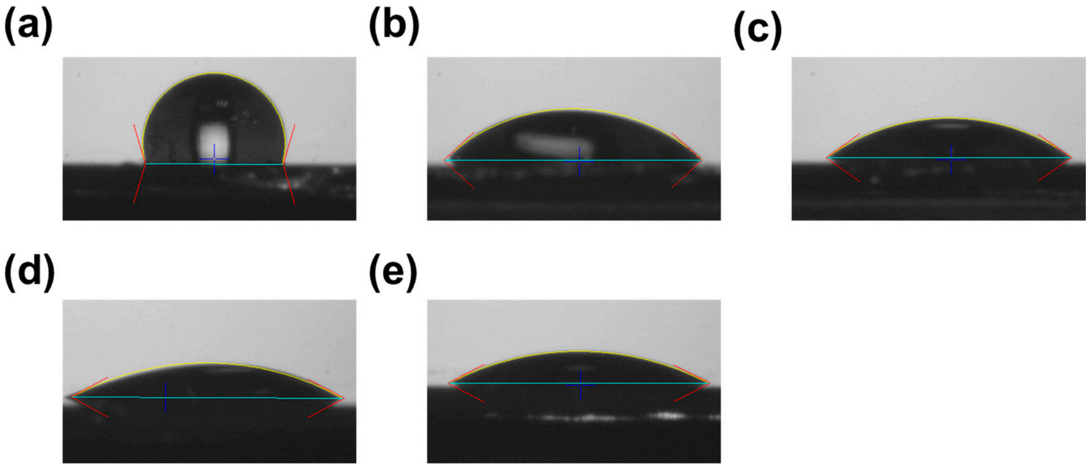

Figure S9. Contact angle images of DPP-DTT with varying weight ratios of azide after development. (a) DPP-DTT-0, (b) DPP-DTT-10, (c) DPP-DTT-50, (d) DPP-DTT-100, and (e) DPP-DTT-150.

Table S2. Summary of contact angle analysis results.

sample name	contact angle (°)	surface energy (mN m^{-2})
DPP-3T-0	110±3	11.75
DPP-3T-10	99±3	12.71
DPP-3T-50	96±3	14.78
DPP-3T-100	86±2	20.53
DPP-3T-150	79±4	25.47
DPP-DTT-0	106±2	9.505
DPP-DTT-10	43±2	54.34
DPP-DTT-50	36±2	59.65
DPP-DTT-100	28±3	64.0
DPP-DTT-150	28±2	64.66

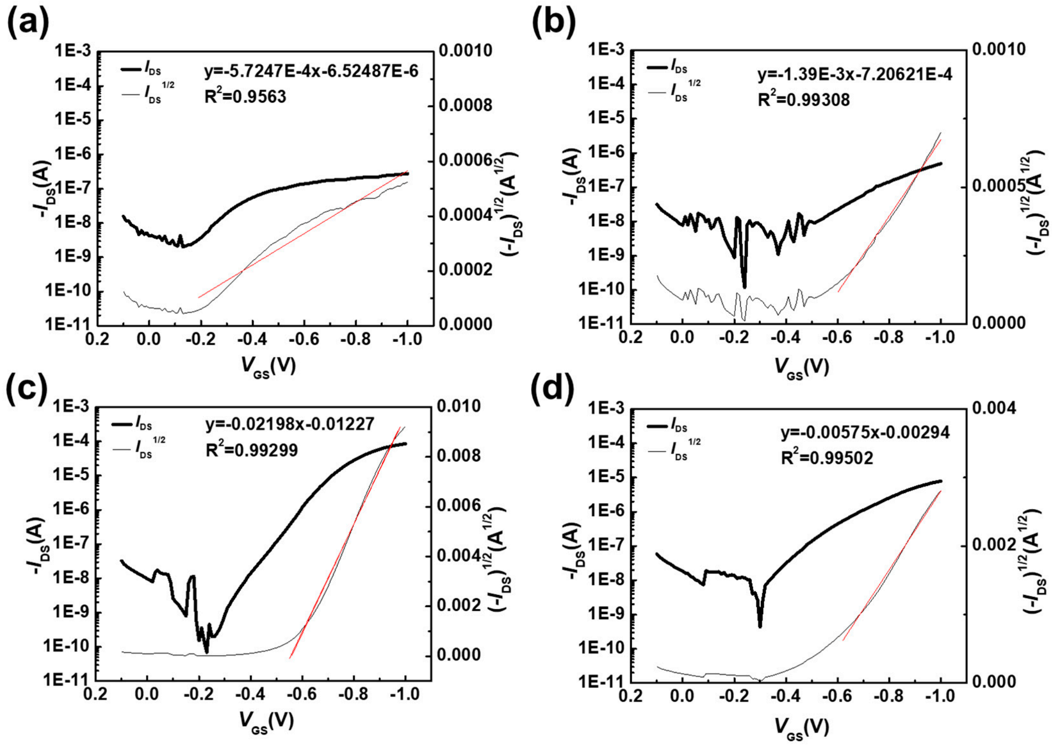

Figure S10. Transfer curve and $I_{DS}^{1/2}$ and V_{GS} curves of representative OECT based on (a) DPP-3T-0, (b) DPP-3T-10, (c) DPP-3T-50, and (d) DPP-3T-100. V_{GS} were scanned with step of 0.01 V and delay of 1 s. μC^* product is extracted from the linear portion of $I_{DS}^{1/2}$-versus-V_{GS} curves according to the equation $I_{DS}^{1/2} = (V_{TH} - V_{GS}) \times ((\mathrm{Wd}/2\mathrm{L})\, \mu C^*)^{1/2}$.

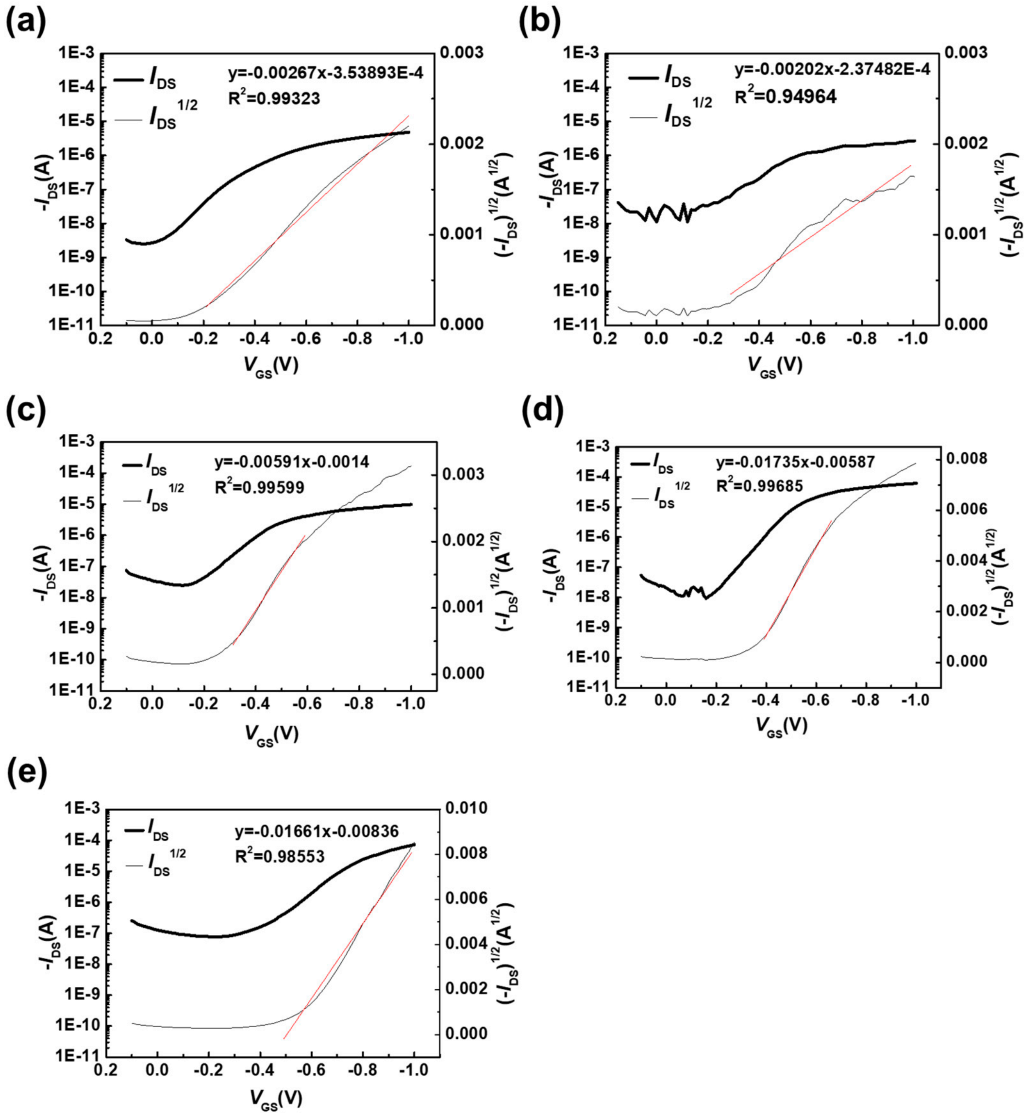

Figure S11. Transfer curve and $I_{DS}^{1/2}$ and V_{GS} curve of representative OECT based on (a) DPP-DTT-0, (b) DPP-DTT-10, (c) DPP-DTT-50, (d) DPP-DTT-100, and (e) DPP-DTT-150. V_{GS} were scanned with step of 0.01 V and delay of 1 s. μC^* product extracted from the linear portion of $I_{DS}^{1/2}$-versus-V_{GS} curves according to the equation $I_{DS}^{1/2} = (V_{TH} - V_{GS}) \times ((\mathrm{Wd}/2\mathrm{L})\, \mu C^*)^{1/2}$.

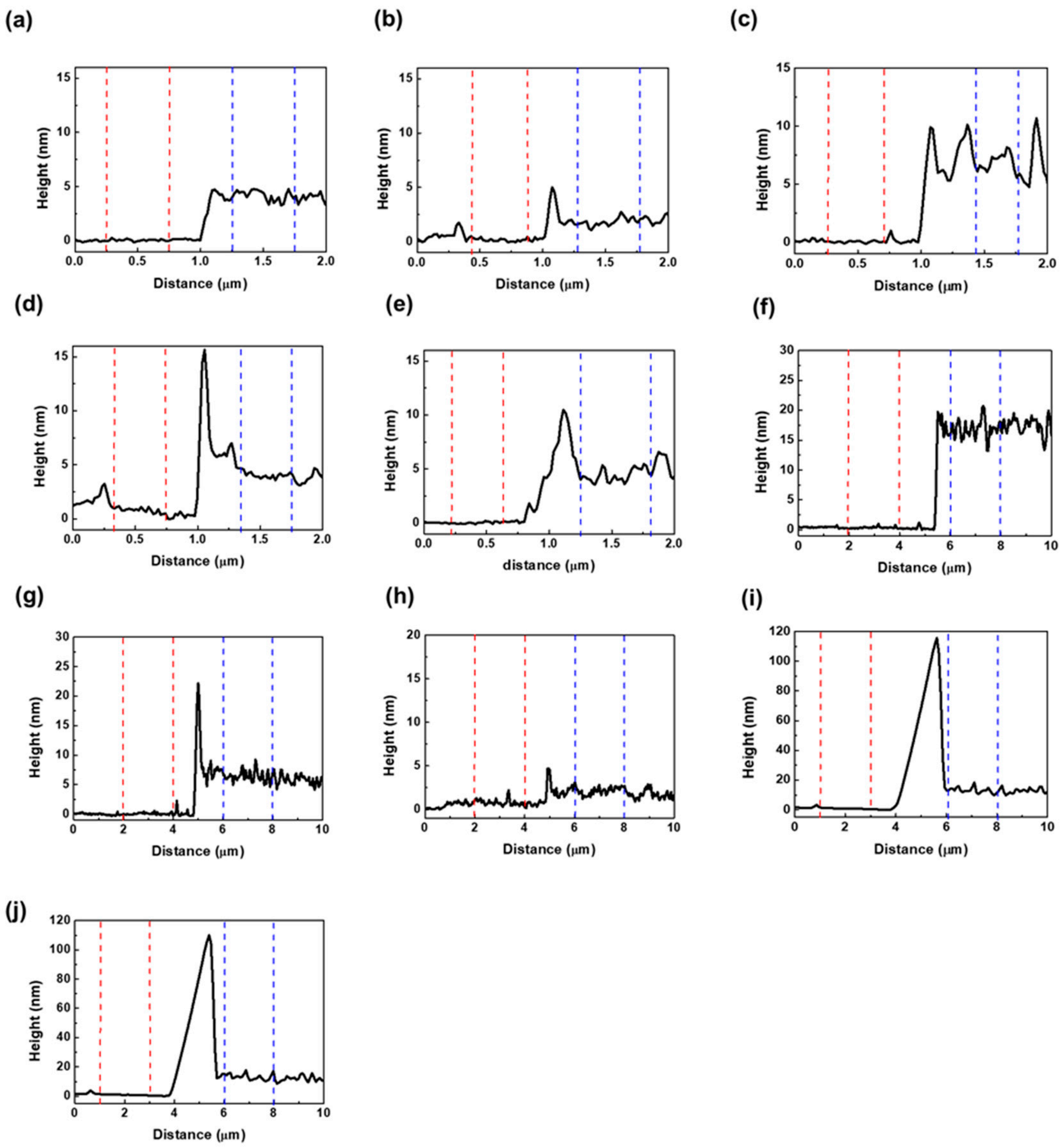

Figure S12. One-dimensional profile from height mode AFM. (a) DPP-3T-0, (b) DPP-3T-10, (c) DPP-3T-50, (d) DPP-3T-100, (e) DPP-3T-150, (f) DPP-DTT-0, (g) DPP-DTT-10, (h) DPP-DTT-50, (i) DPP-DTT-100, and (j) DPP-DTT-150.

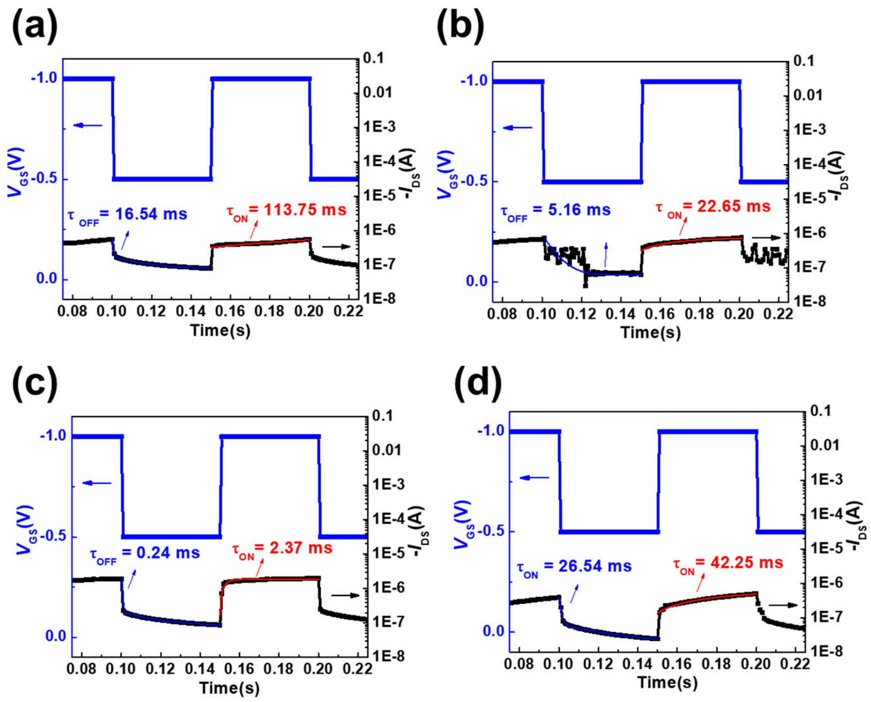

Figure S13. Response time testing of conjugated polymer/azide composite film-based (a) DPP-3T-0, (b) DPP-3T-10, (c) DPP-3T-50, and (d) DPP-3T-100. V_{DS} was fixed at -1 V while V_{GS} was switched from -0.5 V to -1.0 V.

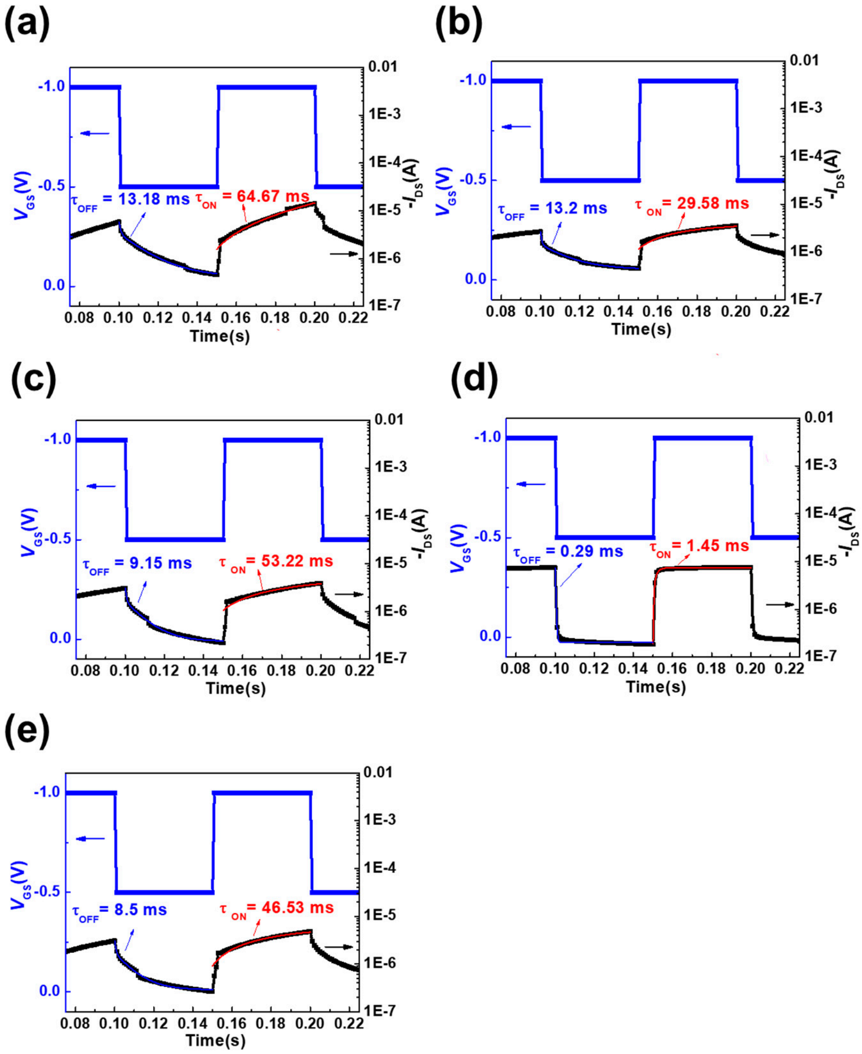

Figure S14. Response time testing of conjugated polymer/azide composite film-based (a) DPP-DTT-0, (b) DPP-DTT-10, (c) DPP-DTT-50, (d) DPP-DTT-100, and (e) DPP-DTT-150. V_{DS} was fixed at -1 V while V_{GS} was switched from -0.5 V to -1.0 V.

Table S3. Overview of the OECT characteristics based on DPP-3T series. All reported electrical performance metrics are the average of 5 independent OECT devices.

	DPP-3T-0	DPP-3T-10	DPP-3T-50	DPP-3T-100
Max. g_m (μS)	0.78 ± 0.53	3.49±1.85	277±155	28.76±13.23
d (nm)	3.56±0.02	3.07±0.01	5.64±0.24	3.49±0.04
[μC^*] ($Fcm^{-1}V^{-1}s^{-1}$)	$(3.92\pm1.17)\times10^{-2}$	$(6.29\pm2.86)\times10^{-1}$	$(8.56\pm3.17)\times10^{1}$	$(4.37\pm1.65)\times10^{1}$
V_{TH} (V)	$(-5.58\pm8.22)\times10^{-1}$	$(-5.18\pm3.21)\times10^{-1}$	$(-5.04\pm0.59)\times10^{-1}$	$(-5.11\pm0.89)\times10^{-1}$
$I_{ON/OFF}$	$(1.73\pm0.51)\times10^{2}$	$(4.10\pm1.51)\times10^{3}$	$(2.75\pm5.34)\times10^{5}$	$(1.81\pm0.71)\times10^{4}$
$\tau_{ON(s)}$	$(1.14\pm0.51)\times10^{-1}$	$(2.27\pm0.78)\times10^{-2}$	$(2.37\pm0.32)\times10^{-3}$	$(4.23\pm1.51)\times10^{-2}$
$\tau_{OFF(s)}$	$(1.65\pm0.67)\times10^{-2}$	$(5.16\pm1.71)\times10^{-3}$	$(2.40\pm0.81)\times10^{-4}$	$(2.65\pm0.97)\times10^{-2}$
C^* ($F\ cm^{-3}$)	422±178	N/A	465±224	N/A
μ ($cm^2V^{-1}s^{-1}$)	$(9.29\pm4.80)\times10^{-5}$	N/A	$(1.84\pm1.12)\times10^{-1}$	N/A

Table S4. Overview of the OECT characteristics based on DPP-DTT series. All reported electrical performance metrics are the average of 5 independent OECT devices.

	DPP-DTT-0	DPP-DTT-10	DPP-DTT-50	DPP-DTT-100	DPP-DTT-150
Max. g_m(μS)	3.95±3.82	10.73±3.77	22.56±6.45	87.30± 45.70	64.36±26.50
d (nm)	16.40±0.10	5.57±0.05	1.44±0.04	11.33±0.21	12.50±0.27
[μC^*] (Fcm^{-1}V^{-1}s-^{1})	(4.35±1.74)×10^{-1}	(9.52±3.56)×10^{-1}	4.99±2.11	26.55±8.85	22.06±7.21
V_{TH} (V)	(-1.20±0.17)×10^{-1}	(-1.18±0.57)×10^{-1}	(-2.37±1.15)×10^{-1}	(-2.03 ± 1.41)×10^{-1}	(-5.03± 1.85)×10^{-1}
I_{ON}/I_{OFF}	(8.15±4.62)×10^{2}	(2.45±1.45)×10^{2}	(1.49±1.78)×10^{2}	(2.48±2.45)×10^{3}	(9.59±7.16)×10^{2}
$\tau_{ON(s)}$	(6.47±0.56)×10^{-2}	(2.96±0.24)×10^{-2}	(5.32±1.16)×10^{-2}	(1.45±0.39)×10^{-3}	(4.65±1.16)×10^{-2}
$\tau_{OFF(s)}$	(1.32±0.65)×10^{-2}	(1.32±0.41)×10^{-2}	(9.15±2.67)×10^{-3}	(2.90±0.81)×10^{-4}	(8.5±2.41)×10^{-4}
C^* (F cm^{-3})	88±21	N/A	N/A	180±34	N/A
μ (cm^{2}V^{-1}s-^{1})	(4.94±2.30)×10^{-3}	N/A	N/A	(1.48±0.57)×10^{-1}	N/A

Table S5. Summary of electrical characteristics for state-of-the-art patterned OECTs.

Active layer	Electrolyte materials	*W*/*L* (μm)	*d* (μm)	I_{ON}/I_{OFF}	μC* product ($Fcm^{-1}V^{-1}s^{-1}$)	τ_{ON} (s)	τ_{OFF} (s)	Maximum g_m (mS)	Patterning resolution (μm)	Refs.
PEDOT:PSS	DN Hydrogel	1800/100	0.15	10^3	317.71 ± 11.61	3.50×10^{-3}	8.10×10^{-3}	*13.2*	*N/A*	*Sci China Mater 2025, 68, 3212–3218 [6]*
DPP-EDOT/PEGDMA	PEGDMA-doped Gel	20/0.06	N/A (vertical OECT)	$(3.30 \pm 0.52)\times10^4$	N/A	$(7.82\pm0.36)\times10^{-3}$	$(1.95\pm0.21)\times10^{-4}$	*53.42± 1.74*	*0.4*	*Proc. Natl. Acad. Sci. U.S.A. 2026, 123 ,e2523877123[7]*
P($g_3$2T-TT)	*ι*-carrageenan (*ι*-CGN)	50/4	5×10^{-3}	10^5	68.06	$2.1\pm0.1\times10^{-4}$	$3.8\pm0.2\times10^{-5}$	*0.11*	*15*	*Adv. Mater. 2025, 37, e09314[8]*
PEDOT:PSS	Electrolyte inks (E003)	14.6 ± 0.4 / 70.3 ± 4.3	≈0.31	$(1.02\pm0.0576)\times10^4$	N/A	1.6×10^{-2}	4×10^{-3}	3.9± 0.36	*15*	*Adv. Mater. Technol.* 2022, *7*, 2200153.[9]
PEDOT:PSS	Poly(diallyldimethylammonium chloride) (E009)	25/200	0.5	2.80×10^4	N/A	2.4×10^{-2}	5.6×10^{-2}	N/A	*25*	*Flex. Print. Electron.* 2022, *7*, 035018.[10]
PEDOT:PSS	Poly(diallyldimethylammonium chloride) (AFI VV009)	N/A	N/A	～ 10^6	N/A	3×10^{-2}	2×10^{-2}	N/A	200	*Nat. Commun.* 2019, *10*, 5053.[11]
DPP-3T-50	PVDF-HFP/ EMIM:TFSI	1000/50	$(5.64\pm0.24)\times10^{-3}$	$(2.75\pm5.34)\times10^5$	85.6±31.7	$(2.37\pm0.32)\times10^{-3}$	$(2.40\pm0.81)\times10^{-4}$	$(277\pm155)\times10^{-3}$	5	This study

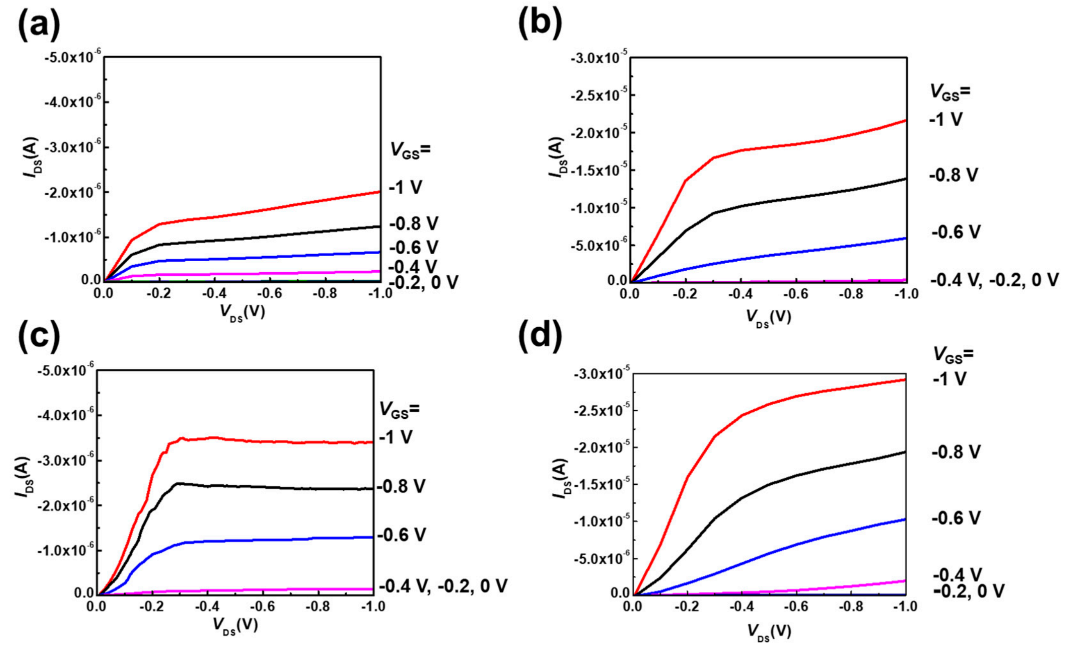

Figure S15. Output characteristics of (a) DPP-3T-0, (b) DPP-3T-50, (c) DPP-DTT-0, and (d) DPP-DTT-100. Sweep delay time：1 s. V_{DS} was scanned with a step of 0.01 V and a delay of 1 s.

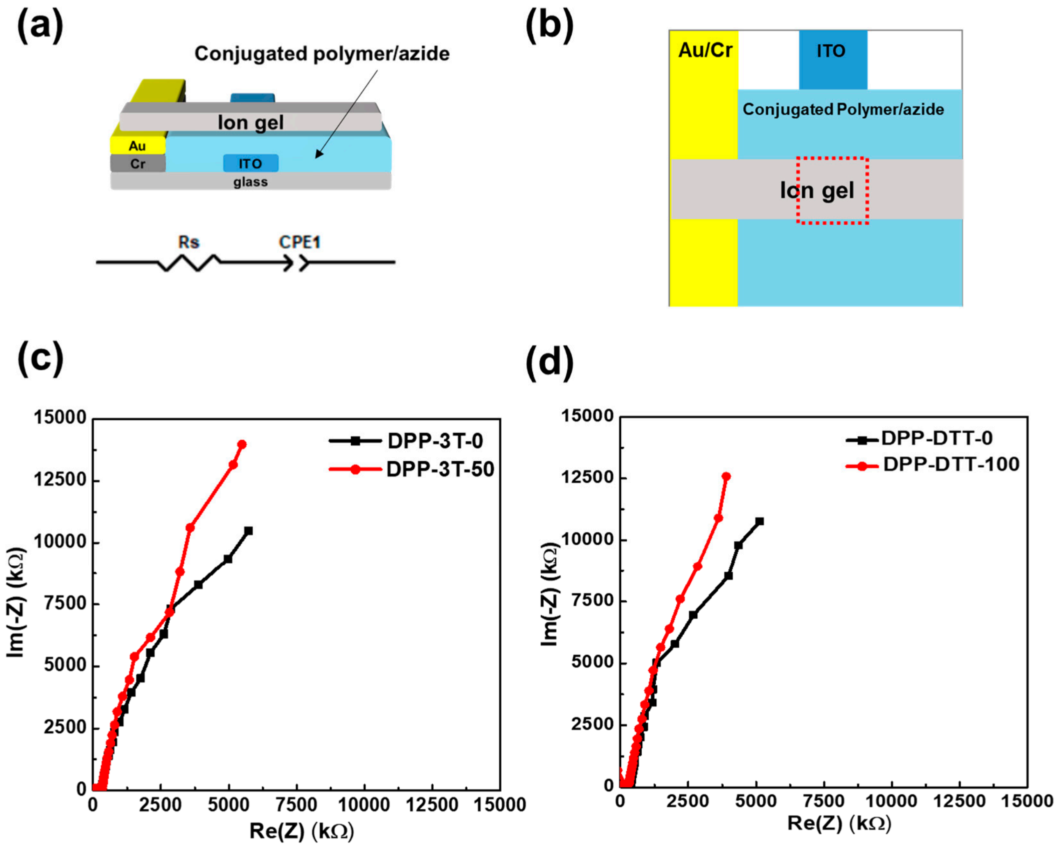

Figure S16. Electrochemical impedance spectroscopy (EIS) element (a) schematic diagram and simulated equivalent circuit and (b) top view with red-dotted rectangle demonstrating effective area (0.125 cm^2). Comparison of Nyquist plots of conjugated polymers before and after adding the optimal weight ratio of azide: (c) DPP-3T-0 and DPP-3T-50, (d) pure DPP-DTT-0 and DPP-DTT-100.

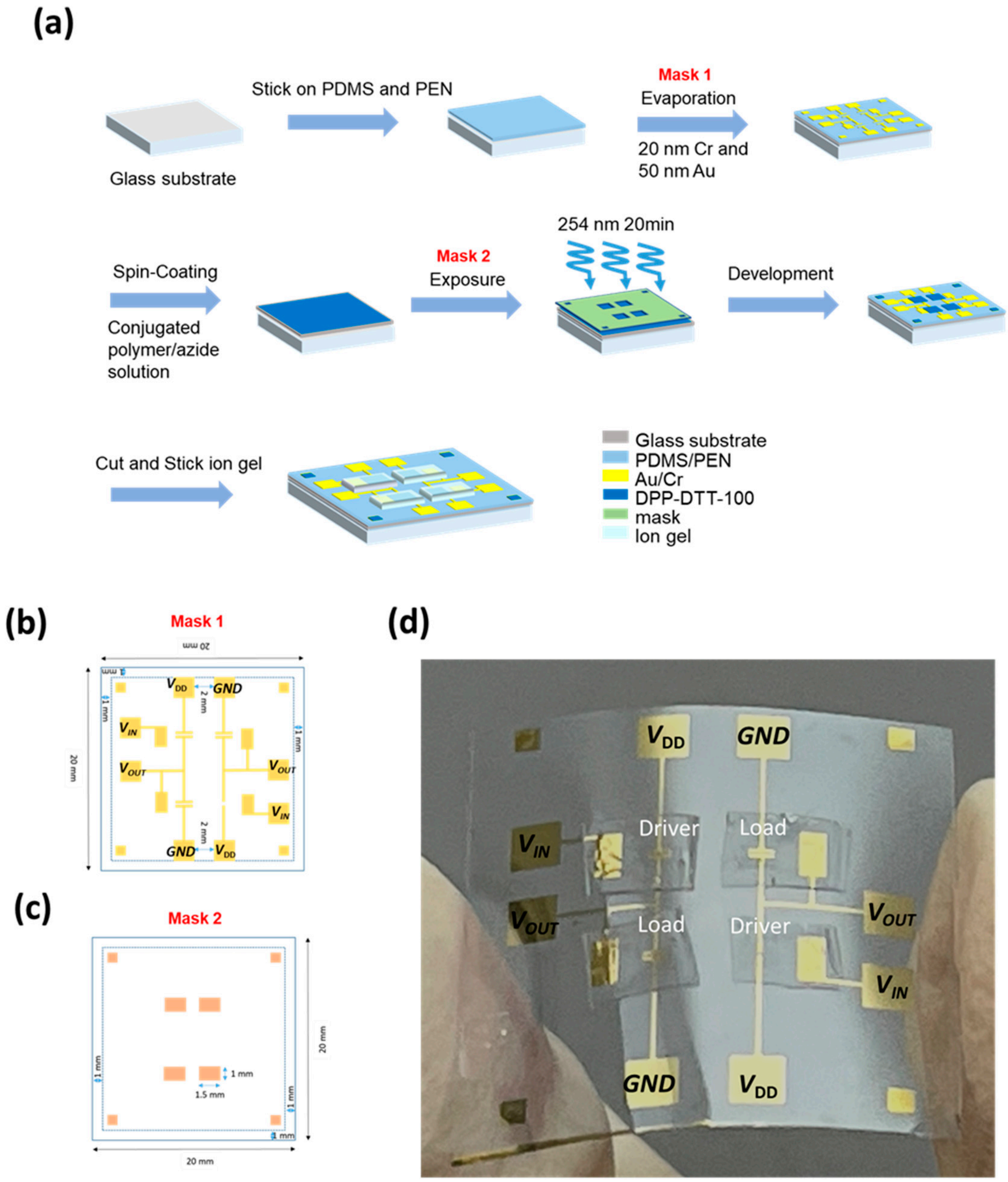

Figure S17. (a) Fabrication process of flexible OECT-based NOT logic gate. (b) The mask for Cr and Au electrodes with K_D/K_L=0.1 and K_D/K_L=1. (c) The mask for patterned conjugated polymer channel. (d) Image of the flexible OECT-based NOT logic gate.

Reference

[1] A. Giovannitti, D.-T. Sbircea, S. Inal, C.B. Nielsen, E. Bandiello, D.A. Hanifi, M. Sessolo, G.G. Malliaras, I. McCulloch, J. Rivnay, Controlling the mode of operation of organic transistors through side-chain engineering, Proc. Natl. Acad. Sci. **2016**, *113*, 12017.

[2] A. Savva, R. Hallani, C. Cendra, J. Surgailis, T.C. Hidalgo, S. Wustoni, R. Sheelamanthula, X. Chen, M. Kirkus, A. Giovannitti, A. Salleo, I. McCulloch, S. Inal, Balancing Ionic and Electronic Conduction for High-Performance Organic Electrochemical Transistors, Adv. Funct. Mater. **2020**, *30*, 1907657.

[3] S.E. Chen, L.Q. Flagg, J.W. Onorato, L.J. Richter, J. Guo, C.K. Luscombe, D.S. Ginger, Impact of varying side chain structure on organic electrochemical transistor performance: a series of oligoethylene glycol-substituted polythiophenes, J. Mater. Chem. A **2022**, *10*, 10738-10749.

[4] D. Ohayon, A. Savva, W. Du, B.D. Paulsen, I. Uguz, R.S. Ashraf, J. Rivnay, I. McCulloch, S. Inal, Influence of Side Chains on the n-Type Organic Electrochemical Transistor Performance, ACS Appl. Mater. Interfaces **2021**, *13*, 4253-4266.

[5] W. Huang, J. Chen, Y. Yao, D. Zheng, X. Ji, L.-W. Feng, D. Moore, N.R. Glavin, M. Xie, Y. Chen, R.M. Pankow, A. Surendran, Z. Wang, Y. Xia, L. Bai, J. Rivnay, J. Ping, X. Guo, Y. Cheng, T.J. Marks, A. Facchetti, Vertical organic electrochemical transistors for complementary circuits, Nature **2023**, *613*, 496-502.

[6] L. Tang, X. Zheng, M. Sun, X. Ren, W. Huang, L. Ye, C. Guo, Y.-X. Wang, W. Hu, Photopatternable gel electrolytes for stretchable solid-state organic electrochemical transistors, Science China Materials **2025**, *68*, 3212-3218.

[7] L. Gao, Y. Cho, R. Wang, Y. Yao, D. Zhang, Y. Wang, Y. Cheng, Y. Wang, Q. Ma, I.

Duplessis, S. Chung, E. Wilson, K. Cho, B. Wang, L. Li, J. Rivnay, H. Wang, J. Yu, T.J. Marks, A. Facchetti, Broadly applicable hydrophilic additive enhances electrochemical transistor function, Proc. Natl. Acad. Sci. **2026**, *123*.

[8] M. Xiong, C.-Y. Yang, J. Ji, A.S. Caravaca, Q. Guo, Q. Li, M.J. Donahue, D. Gao, H.-Y. Wu, A. Marks, Y. Xu, D. Tu, I. McCulloch, P.S. Olofsson, S. Fabiano, A Photo-Patternable Solid-State Electrolyte for High-Performance, Miniaturized, and Implantable Organic Electrochemical Transistor-Based Circuits, Adv. Mater. **2025**, *37*, e09314.

[9] A. Makhinia, K. Hübscher, V. Beni, P. Andersson Ersman, High Performance Organic Electrochemical Transistors and Logic Circuits Manufactured via a Combination of Screen and Aerosol Jet Printing Techniques, Adv. Mater. Technol. **2022**, *7*, 2200153.

[10] M. Zabihipour, P. Janson, M. Berggren, D.T. Simon, P.A. Ersman, I. Engquist, Organic electrochemical transistors manufactured by laser ablation and screen printing, Flex. Print. Electron. **2022**, *7*.

[11] P. Andersson Ersman, R. Lassnig, J. Strandberg, D. Tu, V. Keshmiri, R. Forchheimer, S. Fabiano, G. Gustafsson, M. Berggren, All-printed large-scale integrated circuits based on organic electrochemical transistors, Nat. Commun. **2019**, *10*, 5053.
